# Ecology Drives the Distribution of Specialized Tyrosine Metabolism Modules in Fungi

**DOI:** 10.1093/gbe/evt208

**Published:** 2014-01-02

**Authors:** George H. Greene, Kriston L. McGary, Antonis Rokas, Jason C. Slot

**Affiliations:** ^1^Department of Biological Sciences, Vanderbilt University; ^2^Department of Plant Pathology, The Ohio State University

**Keywords:** pathway evolution, phenolic compound, gene cluster, horizontal gene transfer

## Abstract

Gene clusters encoding accessory or environmentally specialized metabolic pathways likely play a significant role in the evolution of fungal genomes. Two such gene clusters encoding enzymes associated with the tyrosine metabolism pathway (KEGG #00350) have been identified in the filamentous fungus *Aspergillus fumigatus*. The l-tyrosine degradation (TD) gene cluster encodes a functional module that facilitates breakdown of the phenolic amino acid, l-tyrosine through a homogentisate intermediate, but is also involved in the production of pyomelanin, a fungal pathogenicity factor. The gentisate catabolism (GC) gene cluster encodes a functional module likely involved in phenolic compound degradation, which may enable metabolism of biphenolic stilbenes in multiple lineages. Our investigation of the evolution of the TD and GC gene clusters in 214 fungal genomes revealed spotty distributions partially shaped by gene cluster loss and horizontal gene transfer (HGT). Specifically, a TD gene cluster shows evidence of HGT between the extremophilic, melanized fungi *Exophiala dermatitidis* and *Baudoinia compniacensis*, and a GC gene cluster shows evidence of HGT between Sordariomycete and Dothideomycete grass pathogens. These results suggest that the distribution of specialized tyrosine metabolism modules is influenced by both the ecology and phylogeny of fungal species.

## Introduction

Plants produce a diversity of phenolic compounds that serve as defenses against fungal pathogens. Phenolic compounds like stilbenes and flavonoids can be directly toxic to fungi that are unable to metabolize them (Adrian et al. 1997; Fofana et al. 2002). Phenolic compounds can be degraded through the Tyrosine Metabolism pathway (KEGG #00350) to produce simple sugars. The degradation of tyrosine may carry additional costs, however. For example, two human genetic diseases, alkaptonuria and tyrosinemia, are caused by mutations in tyrosine metabolism genes, leading to the accumulation of toxic metabolic intermediates (Jorquera and Tanguay 2001; Peñalva 2001). Interestingly, homologs of genes involved in two parallel tyrosine degradation (TD) pathways are sometimes tightly linked in fungal genomes. This physical co-location of tyrosine metabolism genes is consistent with the view that metabolic genes cluster because natural selection favors co-inheritance and/or balanced expression of enzyme pairs that handle toxic intermediates (Slot and Rokas 2010; Takos and Rook 2012; McGary et al. 2013).

**Fig. 1.—:**
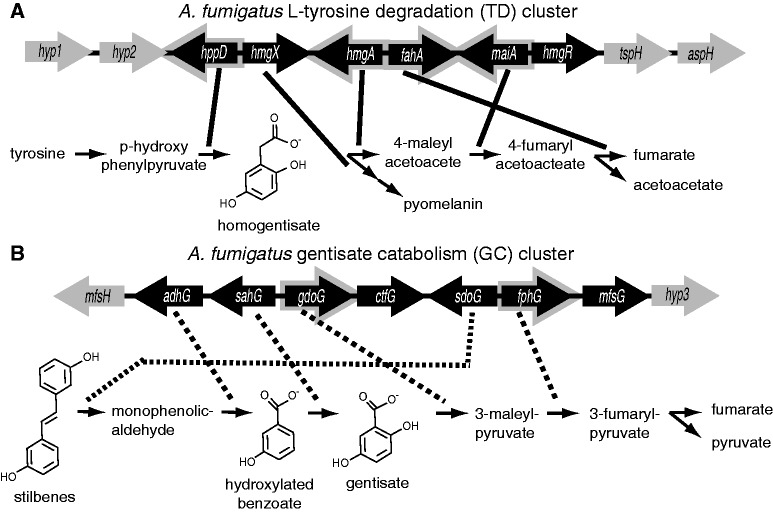
Biochemical pathways for (*A*) the TD gene cluster and (*B*) the putative gentisate catabolism (GC) gene cluster in *A. fumigatus*. TD clusters (indicated by black arrows) include 4-hydroxyphenylpyruvate dioxygenase (*hppD*; Afu2g04200), homogentisate X factor (*hmgX*; Afu2g04210), homogentisate 1,2-dioxygenase (*hmgA*; Afu2g04220), fumarylacetoacetate hydrolase (*fahA*; Afu2g04230), maleylacetoacetate isomerase (*maiA*; Afu2g04240), homogentisate regulatory factor (*hmgR*; Afu2g04262). GC gene clusters include aldehyde dehydrogenase (*adhG*; Afu4g01550), salicylate hydroxylase, (*sahG*; Afu4g01530), gentisate 1,2-dioxygenase (*gdoG*; Afu4g01520), C6 transcription factor (*ctfG*; Afu4g01510), stilbene alpha-beta-dioxygenase (*sdoG*; Afu4g01500), fumarylpyruvate hydrolase (*fphG*; Afu4g01490), and a putative MFS sugar transporter (*mfsG*; Afu4g01480). Solid gray arrows—hypothetical protein 1 (*hyp1*; Afu2g04170), hypothetical protein 2 (*hyp2*;Afu2g04190), TIM17 subunit protein (*tspH*; Afu2g04270), asparaginase (*aspH*; Afu2g04280), myo-inositol transporter (*mfsH;* Afu4g01560), and hypothetical protein 3, a C6 finger domain protein (*hyp3*; Afu4g01470)—are genes that were included in the evolutionary analysis but are not widely conserved. Gray outlined arrows indicate genes encoding enzymes directly involved in tyrosine catabolism. Genes are connected to pathway functions by either solid lines indicating functional evidence for the specific metabolic role or dashed lines indicating putative functions based on enzyme homology. Gene clusters are not drawn to scale.

One of these gene clusters, which is present in the genomes of *Aspergillus* and *Candida* species (Fernández-Cañón and Penalva 1995a; Jorquera and Tanguay 2001; Schmaler-Ripcke et al. 2009; Holesova et al. 2011; Keller et al. 2011), is involved in degrading l-tyrosine through a homogentisate intermediate and consists of four enzymatic steps that convert 4-hydroxyphenylpyruvate to acetoacetate and fumarate (fig. 1*A*). This l-TD gene cluster is also responsible for the formation of pyomelanin in the opportunistic animal pathogen *Aspergillus fumigatus* (Heinekamp et al. 2012). Pyomelanin contributes to disease persistence in some microbial pathogens (Hunter and Newman 2010; Zheng et al. 2013), although such a role has not been demonstrated in *A. fumigatus.* The TD gene cluster is considered a model of hereditary diseases resulting from defects in several of its homologous enzymes in humans (Peñalva 2001).

Through analysis of fungal genome organization (Zhang et al. 2012), we also noted the presence of a separate cluster of genes in a parallel TD pathway in *A. fumigatus*. This gene cluster encodes homologs of five enzymes that are collectively expected to degrade phenolic compounds to fumarate and pyruvate through a gentisate intermediate, and additional proteins putatively involved in phenolic metabolism (fig. 1*B*). We will refer to this as a putative gentisate catabolism (GC) gene cluster.

To better understand the evolution of the TD and GC gene clusters, we investigated their gene order and orientation, distribution, and phylogeny in 214 fungal genomes. Our examination revealed that homologs of the TD and GC gene clusters are present in 50 fungal species spanning multiple genera within the phylum Ascomycota and have a spotty distribution shaped by HGT and gene cluster loss that reflects organisms’ ecological specificity. As a consequence of their physical clustering in fungal chromosomes, complex phenolic metabolism modules may be gained, lost, or modified in a lineage in response to shifting environmental conditions and host–pathogen interactions.

## Materials and Methods

We used two gene clusters from *A. fumigatus* (fig. 1) as queries in a computational pipeline constructed to detect homologous clusters in a database of 214 fungal genomes (supplementary table S1, Supplementary Material online).

The TD query (Schmaler-Ripcke et al. 2009) contained the protein sequences of six characterized genes: 4-hydroxyphenylpyruvate dioxygenase (*hppD*) EC# 1.13.11.27, homogentisate X factor (*hmgX*), homogentisate 1,2-dioxygenase (*hmgA*) EC# 1.13.11.5, fumarylacetoacetate hydrolase (*fahA*) EC# 3.7.1.2, malelylacetoacetate isomerase (*maiA*) EC# 5.2.1.2, and homogentisate regulatory factor (*hmgR*). The TD query also contained two additional sequences from each flanking region: two hypothetical proteins (*hyp1* and *hyp2*), a putative TIM17 subunit protein (*tspH*), and a putative asparaginase (*aspH*) used to assess the degree of the evolutionary conservation of gene cluster boundaries across fungal species.

The *A. fumigatus* gene cluster used as the GC query was first identified by genomic co-location of genes encoding for enzymes in the Tyrosine Metabolism pathway (KEGG #00350) using methods previously described by Zhang et al. (2012). The GC query contained the protein sequences of nine genes: fumarylpyruvate hydrolase (*fphG*) EC# 3.7.2.5, stilbene-alpha, beta-dioxygenase (*sdoG*) EC# 1.13.11.43, a C6 transcription factor (*ctfG*), gentisate 1,2-dioxygenase oxidoreductase (a cupin-fold ring-cleaving dioxygenase, *gdoG*) EC# 1.13.11.4, salicylate hydroxylase (*sahG*) EC# 1.14.13.1, and two sequences from each flanking region (a hypothetical C6 finger domain protein, *hyp3*, a putative MFS sugar transporter, *mfsG*, aldehyde dehydrogenase, *adhG*, and a hypothetical myo-inositol transporter, *mfsH*).

Homologous gene clusters in the fungal database were identified as described previously (Slot and Rokas 2010, 2011; Campbell et al. 2012). Briefly, amino acid sequences similar to the query were identified using BlastP (Altschul et al. 1997; Altschul et al. 2005), retaining sequences with an *e*-value less than 10^−^^4^, that had greater than or equal to 50% amino acid sequence identity to the query sequence, and that were between 50% and 150% the length of the query. Sequences retained after the cutoff were considered to be putatively involved in tyrosine metabolism. rpb2 (RNA polymerase II second largest subunit) sequences were also recovered for construction of a species phylogeny. We restricted downstream analyses of clustering to homologous sequences in clades containing orthologs, recent paralogs of the query sequences, and potential xenologs (homologs derived by horizontal gene transfer [HGT]), based on phylogenetic analyses described later. Homologs were considered clustered if they were separated by at most six intervening genes from a homolog of another gene in the query gene cluster. Gene cluster detection was repeated with the *Exophiala dermatitidis* and *Baudoinia compniacensis* TD loci as queries in order to more accurately evaluate homology among these genomes. Homology among clusters was evaluated first by phylogenetic concordance among constituent protein phylogenies (described later), then by gene content and conservation of synteny. Conservation of the synteny among closely related species differing in the presence of the GC gene cluster was analyzed by multiple genome alignment of the corresponding loci in Mauve 2.3.1 software (Darling et al. 2010). The absence of genes was confirmed by TBlastN of query protein sequences against nucleotide genome assemblies.

Each protein homolog group was aligned with mafft ver. 6.847 (Fernández-Cañón and Penalva 1995b; Jorquera and Tanguay 2001; Katoh et al. 2002; Katoh and Toh 2008; Heinekamp et al. 2012) under default settings. The resulting alignments were manually curated in MacClade (Maddison and Maddison 2003) to remove poorly aligned taxa and then realigned with mafft. Sites in the alignment missing from greater than 30% of taxa were removed with Trimal ver. 1.2 (Capella-Gutierrez et al. 2009). Maximum likelihood analysis was performed in RAxML ver. 7.3.1 (Stamatakis 2006), with 100 bootstrap replicates under the PROTGAMMADAYHOFF model of amino acid substitution, which was determined to be the best fit of all alignments by the ProtTest (Abascal et al. 2005) script implemented in RAxML.

Analyses that forced gene trees to conform to specific topological constraints were performed in RAxML using the same model parameters. TD and GC phylogenies were constrained to contain clades consisting solely of all Dothideomycetes sequences, all Eurotiomycetes sequences, or all Sordariomycetes sequences (supplementary fig. S1, Supplementary Material online). Likelihood scores of optimal and constrained topologies were compared using the Approximately Unbiased test implemented in the Consel program (Shimodaira 2002). To control for long-branch attraction artifacts, we used the tree-independent method implemented in TIGER (http://bioinf.nuim.ie/tiger/, last accessed January 7, 2014) to classify character evolution rates (Cummins and McInerny 2011) and performed additional maximum likelihood analyses, which excluded the fastest two of ten rate partitions.

Because some homologs of gentisate dioxygenase (gdoG) are known to use salicylate preferentially as a substrate, we sought to better estimate the specificity of fungal gdoG by comparing known function-relevant residues across a set of fungal and a set of bacterial sequences. gdoG amino acid sequences from each set were aligned with mafft and imported into WebLogo 3.3 (Crooks et al. 2004) to determine the extent of conservation and similarity of amino acid residues. Residues of interest (Matera et al. 2008; Ferraroni et al. 2012; Ferraroni et al. 2013) were then compared in the two alignments and with a salicylate-cleaving homolog in *Pseudaminobacter salicylatoxidans* (Matera et al. 2008).

## Results

### Three Types of TD Gene Clusters Are Found in Dothideomycetes and Eurotiomycetes

**Fig. 2.—:**
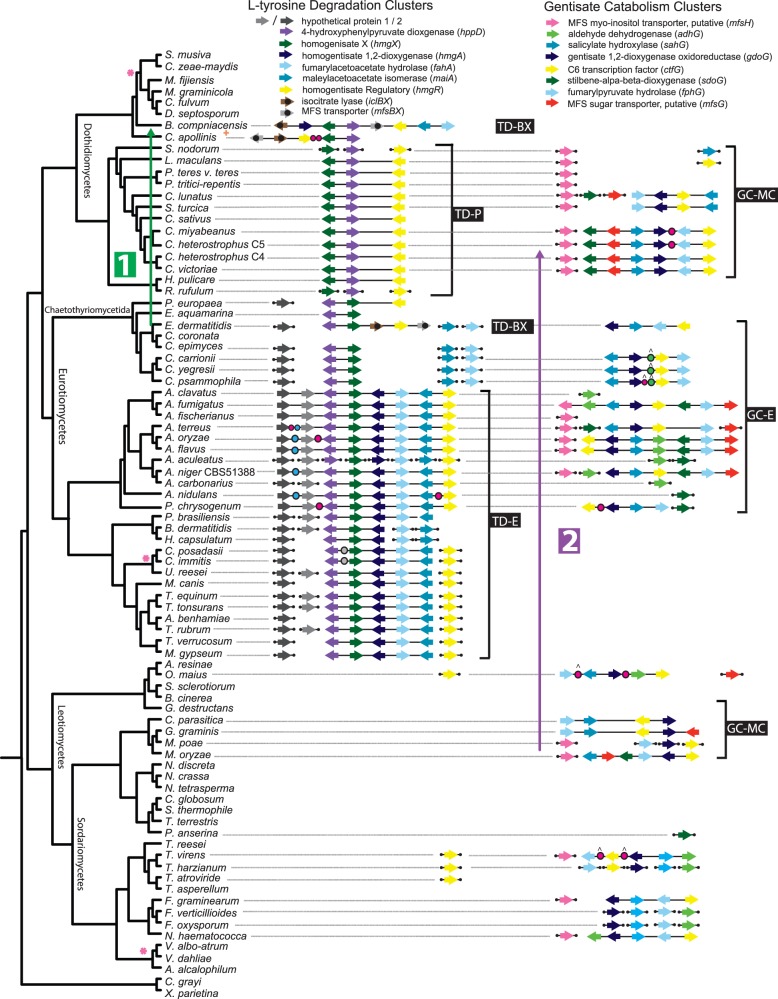
Distribution of two specialized tyrosine metabolism gene clusters in Ascomycete fungi. The rpb2 phylogeny (left panel) depicts species relationships among the lineages beside homologs of the l-tyrosine degradation (TD) gene clusters (middle panel) and gentisate catabolism (GC) gene clusters (right panel). Gene colors indicate homology with the query genes in *A. fumigatus*; absence of gene arrows for taxa denotes absence of these orthologs from their genomes. Clustering is indicated by solid lines connecting genes. Homologs that are not clustered are indicated by detached line segments. Specific gene cluster types defined first by clades in gene trees then by shared synteny are indicated with brackets. Genes with black dots in *B. compniacensis* and *E. dermatitidis* TD gene clusters were not part of the initial query. The first of these (brown) is isocitrate lyase (*iclBX*), and the second (gray) is an MFS transporter gene (*mfsBX*). The pink asterisk denotes that a homolog of MFS myo-inositol transporter of the GC gene cluster was detected for all species in the indicated clade. Dots in clusters represent other intervening genes not found in the *A. fumigatus* query: magenta dots represent unique intervening genes, and all other colored dots represent homologs (i.e., the blue dots in *Aspergillus* clade are homologous). Dots with carets above them indicate the presence of 2–6 intervening genes. Labeled arrow 1 indicates a horizontal transfer of a TD-BX gene cluster from a relative of *E. dermatitidis* (Eurotiomycetes) to *B. compniacensis* (Dothideomycetes) and labeled arrow 2 indicates a horizontal transfer of a GC-MC gene cluster from a relative of *Magnaporthe* spp. (Sordariomycetes) to Pleosporineae (Dothideomycetes).

**Fig. 3.—:**
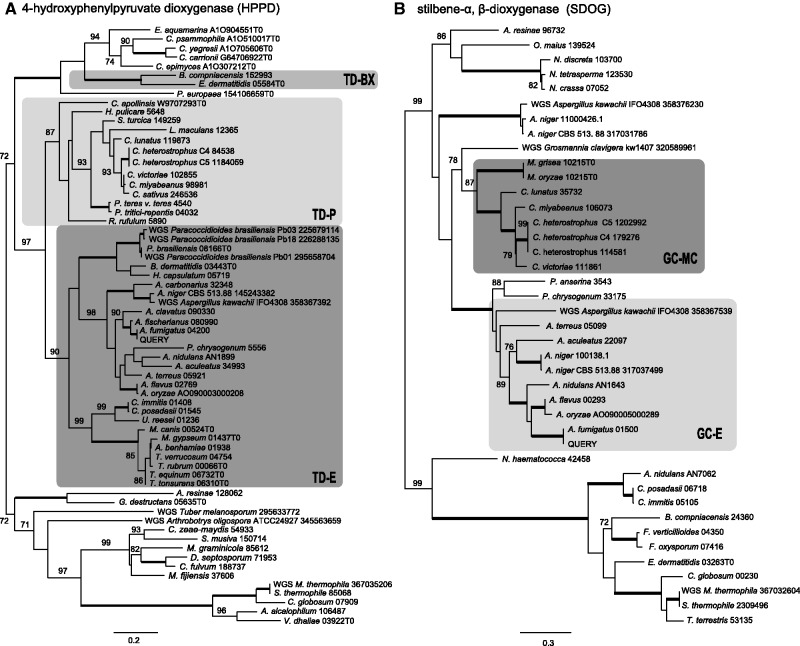
Phylogenetic trees of (*A*) 4-hydroxyphenylpyruvate dioxgenase (hppD) encoded in the TD gene cluster and (*B*) stilbene-α, β-dioxygenase (sdoG) encoded in the gentisate catabolism (GC) gene cluster. The ortholog group for each cluster type is bounded by a shaded box. Support values represent percentage bootstrap support (out of 1000) under maximum likelihood. Only values greater than or equal to 70% are shown. Thickened black lines indicate nodes with 100% support.

We identified 38 TDs (fig. 2) distributed among Dothideomycete and Eurotiomycete genomes. Examination of these clusters enables their division into three general types (TD-E, TD-P, and TD-BX) defined by overall concordance of specific clades in the protein phylogenies (fig. 3*A* and supplementary fig. S2*A–H*, Supplementary Material online) that correspond to the gene content and order of these types. The TD-E type consists of gene clusters that closely match the query gene cluster in gene content and order and which are found in Eurotiomycetes (excluding *E. dermatitidis* and other Chaetothyriomycetida). Clustering of *hppD*, *hmgX*, *hmgA*, *fahA*, and *maiA* in this order is highly conserved in *Penicillium* and *Aspergillus*, and clustering of *hmgR* is additionally well conserved in *Aspergillus*. *hmgR* orthologs are present within most genomes in Eurotiomycetes even when not clustered. The TD-P type consists of gene clusters that contain three genes (*hmgX*, *hppD*, and *hmgR*) differently ordered than their counterparts in TD-E gene clusters and is found exclusively in the Pleosporomycetidae (Dothideomycetes). The TD-BX type also consists of gene clusters that contain *hmgX*, *hppD*, and *hmgR*, but in a different order, and is found only in one Eurotiomycete, *E. dermatitidis*, and one Dothideomycete, *B. compniacensis*. TD-BX type gene clusters share two additional proteins not otherwise associated with TD gene clusters, isocitrate lyase (*iclBX*) and an MFS transporter (*mfsBX*). Furthermore, *fahA* and *maiA* orthologs are not part of the TD-BX gene cluster found in *E. dermatitidis*. *hppD*, *hmgX*, and sometimes *hmgR* are found clustered in the greater Chaetothyriomycetida (Eurotiomycetes), which contains *E. dermatitidis.* A similar TD cluster including *iclBX* and *mfsBX* in *Coniosporium apollinis* has similar gene content to the *E. dermatitidis* TD-BX cluster. Gene order is not highly conserved between the two TD-BX clusters.

### GC Gene Clusters Have a Spotty Distribution in Three Classes of Fungi

We identified 23 GC gene clusters (fig. 2) spottily distributed among genomes from Dothideomycetes, Eurotiomycetes, and Sordariomycetes through the concordance of topologies among protein phylogenies (fig. 3*B* and supplementary fig. S2*J–T*, Supplementary Material online). We were able to identify two general types (GC-E and GC-MC) defined by general concordance of specific clades in the protein phylogenies that correspond to the gene content. In protein phylogenies, these clades are usually nested among paraphyletic Sordariomycete sequences and sequences from *Oidiodendron maius* and *E. dermatitidis*, which have inconsistent branching patterns. Gene order is not well conserved among these clusters. Four fully conserved genes in these gene clusters are *fphG*, *ctfG*, *gdoG*, and *sahG*. The GC-E type consists of gene clusters most closely matched in gene content to the query cluster and is found only in *Aspergillus* and *Penicillium*. *sdoG*, *adhG*, and *mfsG* are variably clustered in GC-E. The GC-MC type consists of gene clusters found in divergent lineages in Pleosporaceae (Dothideomycetes) and Sordariomycetidae (Sordariomycetes). *sdoG* and *mfsG* are variably found in Pleosporaceae and Sordariomycetidae GC-MC gene clusters, but *adhG* is not found in gene clusters in these groups. *Exophiala dermatitidis* and *O. maius* display wide variation in their placement in tree topology. The presence of closely related paralogous gene clusters (supplementary fig. S3, Supplementary Material online) in *O. maius* and *E. dermatitidis* suggest that gene cluster duplication has also played a role in diversification of GC gene clusters. Comparisons of *gdoG* amino acid residues among fungi and bacteria (supplementary table S2 and fig. S4, Supplementary Material online) indicate structural diversity, which may allow relaxed substrate specificity and cleavage of salicylate and other benzoate derivatives in fungi.

### Phylogenetic Analyses Reveal Conflict between Gene Clusters and the Species Phylogeny

The gene trees from hmgR, hppD, hmgA, fahA, and hmgX recover the TD-E and TD-P clades (those of maiA, hyp1, and hyp2 lack TD-P clade sequences), and within these two clades, branching order is consistent with the species phylogeny. The recovery of a *B. compniacensis* and *E. dermatitidis* (BX) clade in four TD gene trees, however, conflicts with a phylogeny of Ascomycota. fahA and hmgA trees do not recover a BX clade but are consistent with a hypothesis that *B. compniacensis* homologs are monophyletic with the greater Chaetothyriomycetida. In addition to three TD genes inferred by comparison with the query gene cluster, these genomes share two other genes in their TDs, *iclBX* and *mfsBX*. The conflict between these gene trees and the species phylogeny is confounded by sequences from a cluster containing both genes in *Rhytidhysteron rufulum*, which show alternative branching orders. *Baudoinia compniacensis* is the only Dothideomycete in which *hmgA* and *maiA* orthologs or xenologs were detected. TD cluster sequences from *C. apollinis*, a related Dothideomycete, group with other Dothideomycetes and are not in conflict with a species phylogeny. Synteny of *hppD*, *hmgX*, and *hmgA* and of *fahA* and *maiA* is shared between *B. compniacensis* and gene clusters of the TD-E type but not of the TD-P type. Approximately Unbiased (AU) tests, in which constrained topologies (enforcing either a Dothideomycete clade or a Eurotiomycete clade) were compared with the optimum topology (BX clade), rejected monophyly of both classes (*P* < 0.05) in *hppD*, *hmgR*, *hmgX*, and *maiA* (table 1). The TD-BX clade was also supported by analyses of hmgR, iclBX, and hppD and *B. compniacensis* grouped with Chaetothyriomycetida in analyses of maiA after exclusion of the fastest evolving characters, suggesting that these results are not long-branch attraction artifacts.

**Table 1:** Topology Tests Fail to Reject Horizontal Transfer

Protein	Optimum Clade	Clade Enforced	*P* Value (AU Test)
hppD	TD-BX	Dothideomycetes	3e−06
		Eurotiomycetes	1e−11
hmgR	TD-BX	Dothideomycetes	1e−05
		Eurotiomycetes	4e−04
hmgX	TD-BX	Dothideomycetes	1e−03
		Eurotiomycetes	7e−03
fahA	TD-BX	Dothideomycetes	>5e−02
		Eurotiomycetes	5e−02
maiA	TD-BX	Eurotiomycetes	3e−05
sdoG	GC-MC	Sordariomycetes	3e−33
sahG	GC-MC	Sordariomycetes	3e−48
gdoG	GC-MC	Sordariomycetes	3e−03
ctfG	GC-MC	Sordariomycetes	4e−20
fphG	GC-MC	Sordariomycetes	5e−09

Protein phylogenies of six genes in the GC cluster support a GC-MC clade. Branching order supports monophyly of *Magnaporthe* and Pleosporineae GC-MC gene clusters. The placement of a *sahG* ortholog from *Stagonospora nodorum* and a *ctfG* ortholog from *Leptosphaeria maculans* as outgroups of the clustered orthologs suggests that the GC-MC gene cluster was originally found more broadly in Pleosporineae but was subsequently subject to repeated degradation and loss events. Five protein phylogenies (sbdG, sahG, gdxG, ctfG, and fphG) had the required taxon sampling to make them amenable to constrained analyses, which forced a Sordariomycetes clade, and all of these rejected monophyly. There was additional support for a GC-MC cluster clade, albeit very weak, from shared synteny between the two lineages, which included the conserved convergent transcription of gdoG and *fphG* among GC-MC type gene clusters. The presence of transposable elements in the flanks of the *Cochliobolus* species was notable due to the ability of these elements to mobilize DNA, which raises the hypothesis that the unexpected distribution of these genes may be explained by HGT.

### Presence of GC Gene Clusters Is Variable among Closely Related Species

In the *Aspergillus* section *Nigri* (Eurotiomycetes), *A. aculeatus* and *A. carbonarius* lack a GC-E type gene cluster, while such a gene cluster is present in *A. niger*. Similarly, *A. clavatus* (section *Clavati*) and its close relative *Aspergillus fischerianus* (section *Fumigati*) lack a GC-E gene cluster, while *A. fumigatus* (section *Fumigati*), the sister taxon of *A. fischerianus*, has a full GC-E gene cluster. Similarly, *A. nidulans* (section *Nidulantes*) lacks a GC-E gene cluster, but *Penicillium chrysogenum* has a partial (4-gene) GC-E gene cluster and two additional unclustered homologs, suggesting that these genes are ancestral in mitosporic Trichocomaceae but were later lost. Mauve alignments of the GC locus in *A. fischerianus* and *A. fumigatus* (supplementary fig. S5, Supplementary Material online) reveal alternative sets of genes totaling a similar length of ∼12 kb. Orthologs of the alternative gene sets are reciprocally absent in the paired species. Four genomes, *F. oxysporum*, *F. verticillioides*, *T. harzianum*, and *Magnaporthe poae*, do not retain clustering of GC gene cluster homologs but do retain constituent enzymes that bracket putative toxic intermediates in the pathway.

## Discussion

### Conservation of Gene Content and Synteny Suggest Metabolic Phenotypes Conferred by Tyrosine Metabolism Gene Clusters

Genes involved in tyrosine metabolism are widely distributed throughout Ascomycete fungi, a subset of which is found in metabolic gene clusters (supplementary fig. S6, Supplementary Material online). Here, we trace the evolution of two types of gene clusters, which encode processes that overlap background functions of tyrosine metabolism (i.e., they are functionally semi-redundant with noncluster paralogs). The TD gene cluster (Schmaler-Ripcke et al. 2009; Keller et al. 2011) overlaps tyrosine metabolism in the path for homogentisate degradation, and the GC gene cluster overlaps tyrosine metabolism in the path for gentisate degradation. Both of these clusters contain genes conferring specialized or accessory functions beyond those conferred by the core degradation pathways.

Conservation of gene content of these clusters despite extensive general genomic rearrangement circumscribes modular functions (Muto et al. 2013) that are retained by natural selection in fungal lineages. Using these conserved gene cluster constituents as a guide, we propose testable models for the net biochemical functions of these gene clusters (fig. 1). For example, the TD-E gene cluster in *A. fumigatus* has already been characterized to produce pyomelanin as a shunt in the breakdown of tyrosine and also to allow the use of l-tyrosine as a sole carbon source (Schmaler-Ripcke et al. 2009). The TD-BX gene cluster contains many of the same genes as the TD-E gene cluster but also contains isocitrate lyase, which could support downstream carbon retention by these fungi and suggests a role for TD-BX in fungal pathogenesis (Dunn et al. 2009). TD-BX also includes an MFS transporter, which could be involved in transport of simple sugars across the mitochondrial membrane. By analogy with the characterized gene cluster in *A. fumigatus*, the TD-P gene cluster in Dothideomycetes may be involved in pyomelanin production but not in degradation of tyrosine as an energy source. Melanins are pathogenicity factors in fungi, and therefore TD-P could indicate selection focused on this component of the pathway for evasion of host oxidative defenses (Williamson et al. 1998; Langfelder et al. 2003; Karkowska-Kuleta et al. 2009; Youngchim et al. 2011).

In GC gene clusters, we propose a model in which multiple genes are required to generate and degrade gentisate and other benzoate derivatives. In our model of the core GC pathway, a hydroxylated benzoate is converted to gentisate by sahG. sahG is homologous with 3-hydroxybenzoate 6-hydroxylase (mnx2), which is encoded in a GC-like gene cluster in *Candida parapsilosis* where it hydroxylates a benzoate derivative for degradation via gentisate (Holesova et al. 2011). Gentisate is then converted to 3-maleyl pyruvate by gdoG, 3-maleyl pyruvate spontaneously isomerizes to 3-fumarylpyruvate, and finally 3-fumarylpyruvate is converted to fumarate and pyruvate by fphG. A stilbene dioxygenase gene, (*sdoG*) is incompletely but broadly conserved in GC gene clusters. Stilbene dioxygenases convert ethylene-linked phenolic dimers such as pinosylvin and resveratrol into monophenolic aldehydes (Marasco and Schmidt-Dannert 2008). sdoG is similar in sequence to lignostilbene dioxygenase, which preferentially cleaves *trans*-4-hydroxy-3-methoxystilbene (Kamoda et al. 2003) into the monophenolic aldehyde, vanillin. Notably, one of the genes that is conserved across divergent lineages, *adhG*, is predicted to encode an aldehyde dehydrogenase, which may complete the path from stilbene to citric acid cycle intermediates. Relaxed specificities for the 2,5 hydroxylation pattern in gentisate (Fetzner 2012) could allow a broader range of stilbenes or benzoate derivatives to be metabolized by GC pathways (Ferraroni et al. 2012; Ferraroni et al. 2013). The conserved sugar transporter in the GC gene cluster may then usher simple sugars into mitochondria for energy production. Similar clusters of homologs of *sahG*, *gdoG*, *fphG*, and maleylpyruvate isomerase are spottily distributed among bacteria expected to be under phenolic stress, such as *Acinetobacter* sp. Strain DR1 (YP_003732082-YP_003732085) (Jung et al. 2010), *Polaromonas naphthalenivorans* strain CJ2 (YP_983363–YP_983366) (Yagi et al. 2009), *Azoarcus* sp. strain BH72 (YP_933924–YP_933927) (Krause et al. 2006), and *Cupriavidus necator* JMP134 (YP_300048–YP_300051) (Lykidis et al. 2010), further supporting the functional association of these genes.

### A Spotty Distribution of Specialized Tyrosine Pathway Clusters Is Explained by Overlapping Ecology

The spotty distribution of specialized tyrosine metabolism clusters is inconsistent with fungal phylogeny. Distantly related species share similar clusters, while close relatives with different ecologies differ in the presence of clusters. This spotty distribution is best exemplified by the fact that the locus containing the GC gene cluster in *A. fumigatus* is occupied by an alternative set of genes in its very close relative *A. fischerianus*. The genes in this locus are reciprocally absent in *A. fumigatus* and *A. fischerianus* suggesting either recent replacement of the GC gene cluster by HGT or a gene cluster polymorphism in this locus (Gibbons et al. 2012; Zhang et al. 2012). *Aspergillus fischerianus* does not readily decay grasses like its close relative *A. fumigatus*, which is often a primary agent of grass silage spoilage (Aragon et al. 2011), suggesting that the difference in content at this locus might reflect the different selective pressures exerted on the two species.

In general, the robustly supported observed discordances between gene tree topologies and the species phylogeny can be explained by either HGT of gene clusters or by the independent maintenance of an ancient duplicate gene cluster in specific lineages and its loss in all others. For example, the clade formed by genes in TD-BX gene clusters suggests either HGT from Chaetothyriomycetida to an ancestor of *B. compniacensis* or retention of an ancient duplicate cluster by *B. compniacensis* and *E. dermatitidis*, but not by other Dothideomycetes or Chaetothyriomycetida genomes. We favor the hypothesis of HGT in this case due to the high number of gene cluster losses required to reconcile the gene trees with the conflicting species phylogeny under the alternative hypothesis (see also Khaldi et al. 2008). Similarly, the phylogeny of GC genes is best explained by HGT of the GC-MC cluster between *Magnaporthe* and the clade containing *Cochliobolus* spp*.* There is an intriguing phenotypic overlap between divergent species that is not found among all species of their respective classes present in our data set. Both *E. dermatitidis* (like other Chaetothyriomycetida) and *B. compniacensis* have constitutively melanized cell walls and thrive in extreme environments. *Exophiala dermatitidis*, although most known as a human pathogen acquired in bathhouses in Southeast Asia, is most readily cultured from creosote-treated railroad ties (Sudhadham et al. 2008). The preservative effect of creosote on exposed wood is due in part to antifungal phenolic compounds (Kim et al. 2010). *Baudoinia compniacensis* is most commonly isolated from exterior surfaces near alcohol distilleries (Scott et al. 2007). Both of these species are thermotolerant and resistant to environmental exposure. Aside from human environments, fallen fruit has been postulated as a natural reservoir for both of these fungi (Scott et al. 2007; Sudhadham et al. 2008). Sharing niches under oxidative stress may have provided an opportunity for the ancestors of *B. compniacensis* and *E. dermatitidis* to engage in HGT.

Acquisition of the TD-BX gene cluster may have benefitted *B. compniacensis* by facilitating additional protective melanization and assimilation of carbon. Two genes (*hmgR* and *hmgX*) that may have been transferred with the TD-BX gene cluster facilitate a metabolic shunt for the production of pyomelanin (Heinekamp et al. 2012) and appear to be ancestrally present in TD gene clusters. This shunt may benefit *B. compniacensis* by converting high concentrations of toxic phenolic compounds to pyomelanin, which protects cells from oxidation damage (Rodriguez-Rojas et al. 2009). The inclusion of isocitrate lyase in the transferred gene cluster suggests these species are able to directly assimilate fixed carbon from the breakdown of phenolics (Dunn et al. 2009).

The GC gene cluster variants in this study are likely to facilitate metabolic specialization by prepending one or two additional metabolic steps upstream of ring cleavage. These clusters all encode sahG, which may transform benzoate derivatives for degradation by gdoG. Salicylate and gentisate are plant pathogen-response hormones with antifungal activity (Dempsey et al. 2011; Qi et al. 2012). Biotrophic and endophytic fungi may require resistances to these compounds to maintain stable infections of plant hosts (Ambrose and Belanger 2012). The fungi in this analysis that can live endophytically in plants are often found with GC gene clusters that are expected to degrade benzoate derivatives such as gentisate and salicylate. One of them, *Fusarium graminearum*, has also been shown to use salicylate as a carbon source (Qi et al. 2012). These monophenolic compounds are likely to be produced throughout the life history of many plants and consequently favor biotrophs with a mechanism to cope with them. GC gene clusters with *sahG* are found in distantly related endophytic fungi, such as *Fusarium graminearum*, *Trichoderma virens*, and *Oidiodendron maius*, but not in their close relatives with different ecological preferences (*T. reesei—*a wood decayer [Kubicek et al. 2011] and *Amorphotheca resinae—*a hydrocarbon degrader [Seifert et al. 2007]). We hypothesize that this reflects their use in symbiosis-related metabolism.

Stilbenes are antifungal compounds produced by sorghum and other grasses in response to multiple types of pathogenic fungal infections (Brinker and Seigler 1991; Yu et al. 2005, 2008). Consequently, grass pathogens may benefit from alpha-beta dioxygenases, such as sdoG, to degrade stilbenes to monophenolic aldehydes (Adrian et al. 1997; Hipskind and Paiva 2000; Schulze et al. 2005). GC-MC and GC-E types are two GC gene cluster variants that contain *sdoG* orthologs and that are found in grass pathogens (*Magnaporthe* spp. and *Cochliobolus* spp.) and common spoilage agents of grain (*Aspergillus* spp.), respectively. *Aspergillus* species containing GC-E also elicit production of stilbenes during spoilage of peanut (Sobolev 2013). The distribution of *sdoG*-containing GC cluster variants is more strongly associated with grass colonization than simple linear descent through the species phylogeny, which further suggests that shared ecology provides both the opportunity and selection pressure to drive HGT of gene clusters (Slot and Hibbett 2007; Slot and Rokas 2010; Slot and Rokas 2011; Campbell et al. 2012).

**Fig. 4.—:**
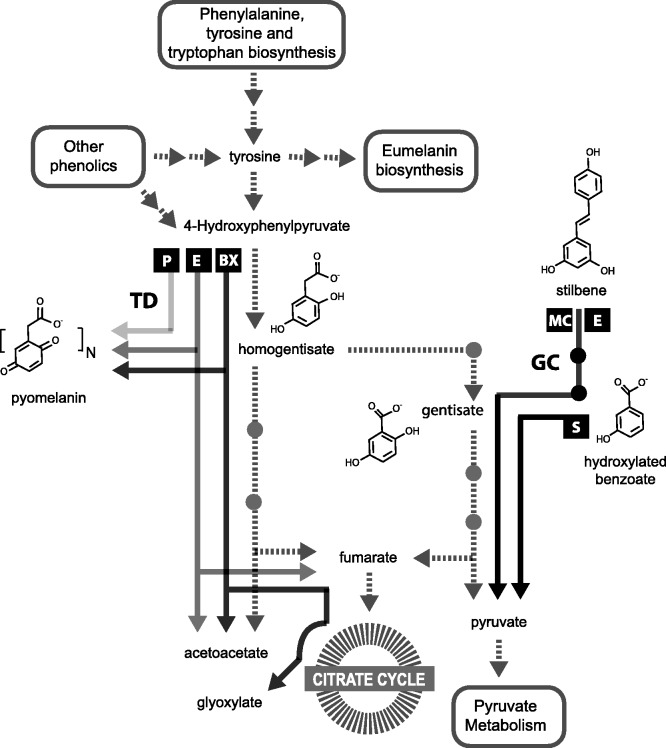
Modular evolution and specialization of pathways in tyrosine metabolism. Basic TD via homogentisate and gentisate is depicted by dashed lines. Intermediate metabolites are indicated by names (and compound structure) or solid points. Metabolic modules and putative metabolic modules encoded by specialized gene clusters that parallel the basic pathways are depicted with solid lines of different shades. The TD-P, TD-E, TD-BX, GC-MC/GC-E, and TD-S (where TD-S is a nonmonophyletic assemblage of clusters expected to degrade benzoate derivative compounds by way of gentisic acid) are indicated from lightest to darkest grayscale.

The ecological pattern to the distribution of specialized tyrosine metabolism gene clusters is facilitated by the physical linkage of the pathways they encode. The core genes involved in tyrosine metabolism are commonly dispersed in the genome, while more specialized genes that function in contexts such as toxin degradation and resistance to host defenses appear to be frequently clustered. Decoupling of specialized functions from core metabolism through gene duplication-induced redundancy (fig. 4) frees specialized genes and gene clusters to adapt to novel usage, while feeding into established metabolic pathways (Zhang et al. 2012). Selection for the maintenance of specialized versions of these pathways may be sporadic and variable due to changing environments and host–pathogen co-evolution, even though the non-clustered core metabolic pathways remain relatively stable. Relaxation of selection from environmental factors explains why specialized pathways are prone to loss during vertical transmission. Gradual loss of the constituent genes may be prevented by selection against the accumulation of toxic intermediate compounds produced by incomplete pathways (McGary et al. 2013), but isolation of these pathways to a specific location weakens their link to the genome by facilitating complete loss in a single event (Slot and Rokas 2010). HGT may counteract the loss of genes that are not under constant selection if they can provide benefit to the organisms that receive them (Lawrence and Roth 1996; Walton 2000). The net benefit of these gene clusters to fungi may be enhanced by additional conserved genes, such as transcription factors and transporters, which contribute to a complete, modular function. In these ways, genomic modularity of complex specialized metabolic pathways enhances the adaptability of receptive fungal genomes to shifts in ecological landscapes.

## Supplementary Material

Supplementary figures S1–S6 and tables S1–S2 are available at *Genome Biology and Evolution* online (http://www.gbe.oxfordjournals.org/).

Supplementary Data
