# Supplementary material for: Ecology Drives the Distribution of Specialized Tyrosine Metabolism Modules in Fungi
Source: Genome Biol Evol. 2014 Jan 2;6(1):121–32. doi: 10.1093/gbe/evt208 (PMC3914699; doi:10.1093/gbe/evt208)
Supplement: Supplementary Data [file supp_6_1_121__index.html]

Ecology drives the distribution of specialized tyrosine metabolism modules in fungi — Ecology Drives the Distribution of Specialized Tyrosine Metabolism Modules in Fungi — Supplementary Data 

# Ecology Drives the Distribution of Specialized Tyrosine Metabolism Modules in Fungi

## Supplementary Data

files

**Files in this Data Supplement:**

- Supplementary Data - pdf file
