# Supplementary material for: Ecology Drives the Distribution of Specialized Tyrosine Metabolism Modules in Fungi
Source: Genome Biol Evol. 2014 Jan 2;6(1):121–32. doi: 10.1093/gbe/evt208 (PMC3914699; doi:10.1093/gbe/evt208)
Supplement: Supplementary Data [file supp_evt208_Greene_etal_Supplement_revised.pdf]

Supplemental Materials

Figure S1. Maximum likelihood analyses of protein alignments in this study. A = *hyp1*, B = *hyp2*, C = *hppD*, D = *hmgX*, E = *hmgA*, F = *fahA*, G = *hmgR*, H = *maiA*, I = *tspH*, J = aspH, K = *mfsG*, L = *fphG*, M = *sdoG*, N = *ctfG*, O = *gdoG*, P = *sahG*, Q = *adhG*, R = *mfsH*, S = *mfsBX*, T = *iclBX,* U = species tree.

Figure S2. Paralogous gentisate catabolism clusters.

Figure S3. Mauve alignments of gentisate catabolism (GC) cluster loci. A. *A. fumigatus* GC cluster and flanking sequences aligned with the *A. fischerianus* homologous locus. B. *A. niger* GC cluster and flanking sequences aligned with the *A. carbonarius* homologous locus. Matching colored histograms correspond to aligned sequence and direction is indicated by position of histograms above or below the horizontal axis.

Figure S4. Distribution and clustering of phenolic metabolism genes in Pezizomycotina fungi. Taxa on y-axis are ordered as in Figure 2 and abbreviated as in Table S1. Cell colors in the matrix indicate the following states: Black = no detected homologs, light blue = single homolog unclustered, dark blue = multiple homologs unclustered, pink = a single homolog, which is clustered, and red = multiple homologs with at least 1 clustered.

Figure S5. Alignment logos of A. bacterial, and B. fungal gentisate 1,2-dioxygenase amino acid sequences. Stack height at each position indicates residue conservation, and relative residue height within each position corresponds to the frequency it is encountered. Known functional positions (see table S2) indicate the corresponding residue in salicylate 1,2 dioxygenase in *Psuedaminobacter salicylatoxidans*.

Table S1. Genomes analyzed in this study.

Table S2. Comparison of function-related residues among gentisate 1,2 dioxygenase homologs in fungi and bacteria.

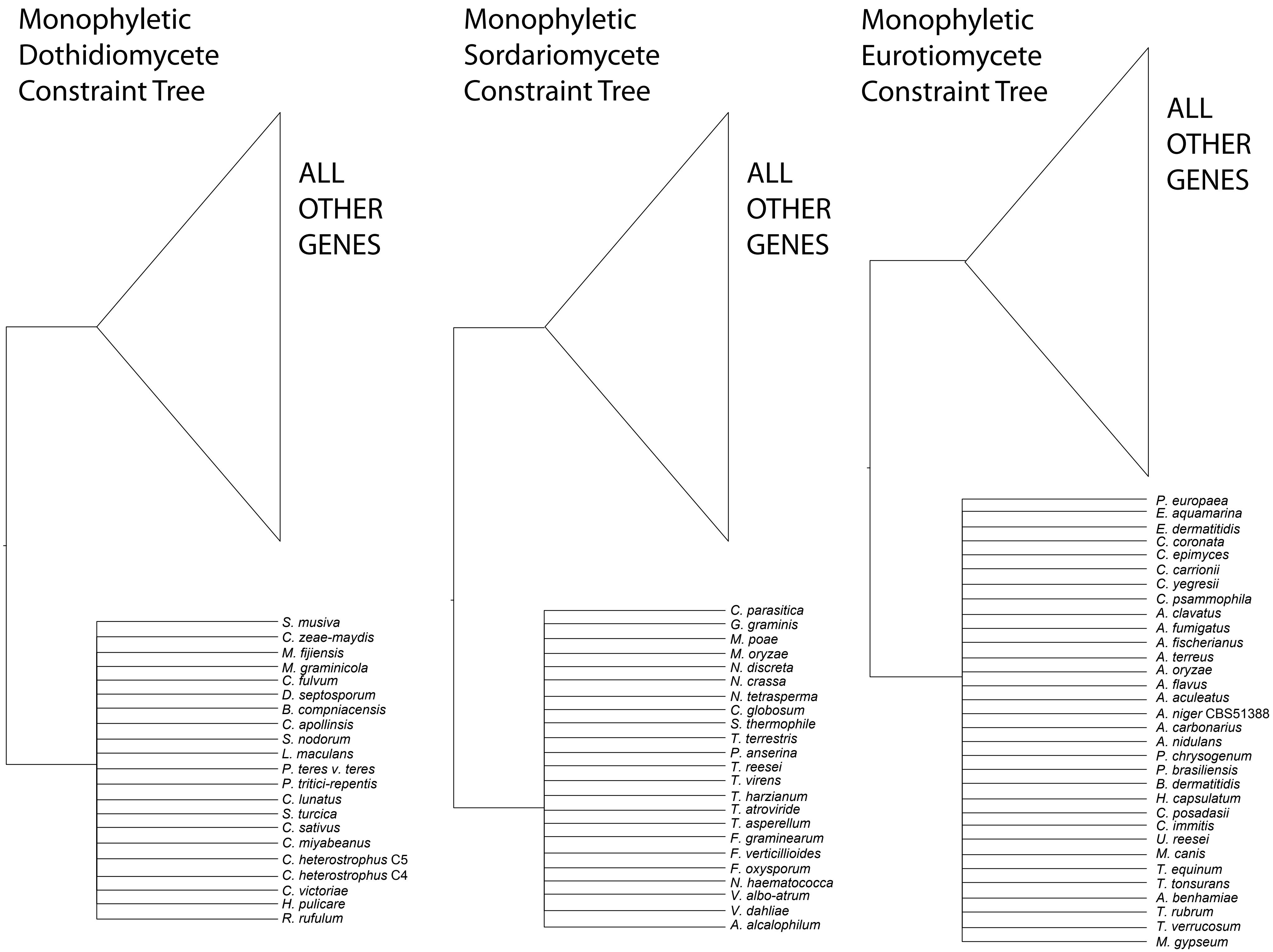

Monophyletic Dothidiomycete Constraint Tree
ALL OTHER GENES
S. musiva
C. zeae-maydis
M. fijiensis
M. graminicola
C. fulvum
D. septosporum
B. compniacensis
C. apollinsis
S. nodorum
L. maculans
P. teres v. teres
P. tritici-repentis
C. lunatus
S. turcica
C. sativus
C. miyabeanus
C. heterostrophus C5
C. heterostrophus C4
C. victoriae
H. pulicare
R. rufulum
Monophyletic Sordariomycete Constraint Tree
ALL OTHER GENES
C. parasitica
G. graminis
M. poae
M. oryzae
N. discreta
N. crassa
N. tetrasperma
C. globosum
S. thermophile
T. terrestris
P. anserina
T. reesei
T. virens
T. harzianum
T. atroviride
T. asperellum
F. graminearum
F. verticillioides
F. oxysporum
N. haematococca
V. albo-atrum
V. dahliae
A. alcalophilum
Monophyletic Eurotiomycete Constraint Tree
ALL OTHER GENES
P. europaea
E. aquamarina
E. dermatitidis
C. coronata
C. epimyces
C. carrionii
C. yegresii
C. psammophila
A. clavatus
A. fumigatus
A. fischerianus
A. terreus
A. oryzae
A. flavus
A. aculeatus
A. niger CBS51388
A. carbonarius
A. nidulans
P. chrysogenum
P. brasiliensis
B. dermatitidis
H. capsulatum
C. posadasii
C. immitis
U. reesei
M. canis
T. equinum
T. tonsurans
A. benhamiae
T. rubrum
T. verrucosum
M. gypseum

A. HYP1

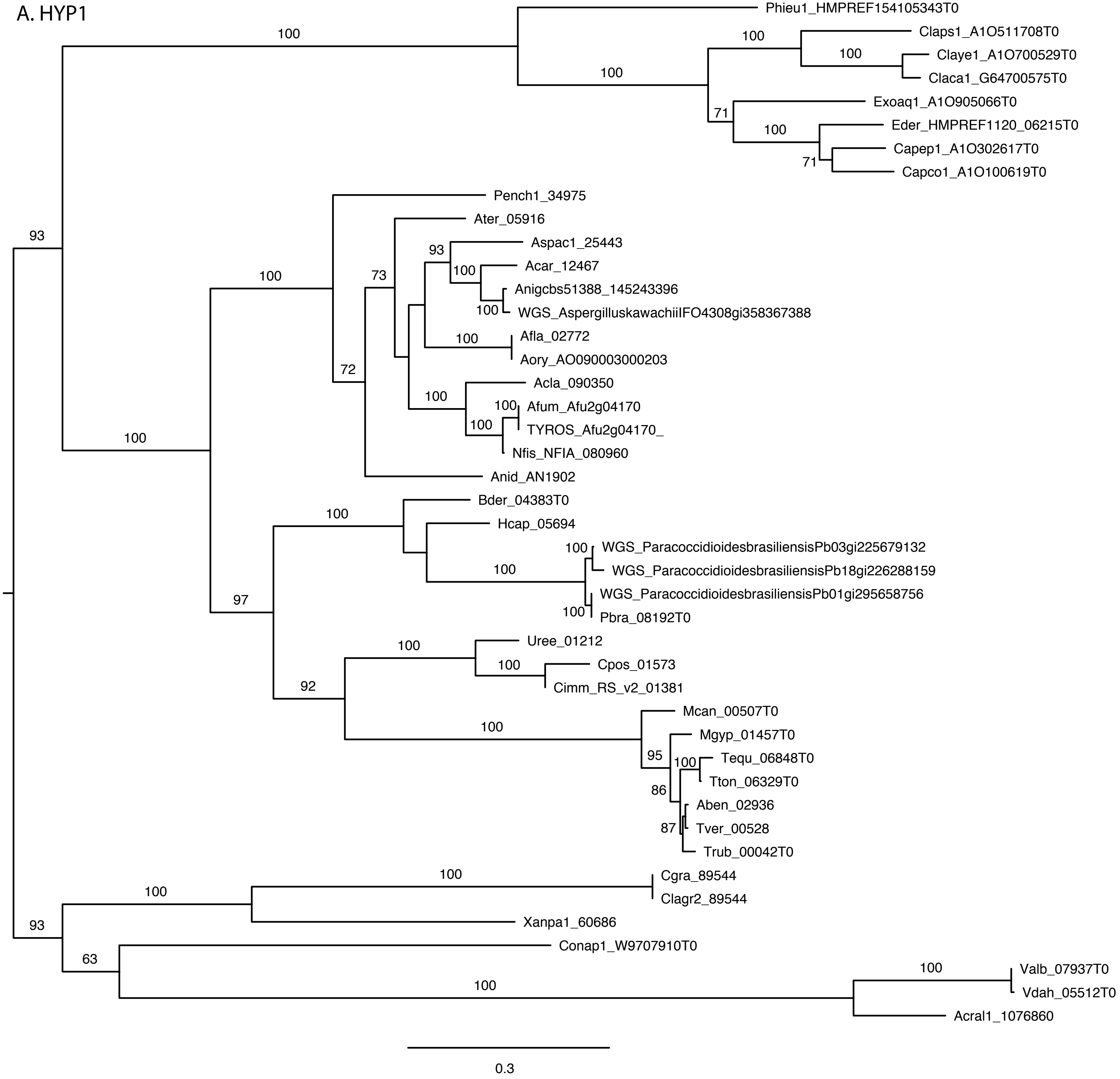

Phieu1_HMPREF154105343T0
Claps1_A1O511708T0
Claye1_A1O700529T0
Claca1_G64700575T0
Exoaq1_A1O905066T0
Eder_HMPREF1120_06215T0
Capep1_A1O302617T0
Capco1_A1O100619T0
Pench1_34975
Ater_05916
Aspac1_25443
Acar_12467
Anigcbs51388_145243396
WGS_AspergilluskawachiiIFO4308gi358367388
Afla_02772
Aory_AO090003000203
Acla_090350
Afum_Afu2g04170
TYROS_Afu2g04170_
Nfis_NFIA_080960
Anid_AN1902
Bder_04383T0
Hcap_05694
WGS_ParacoccidioidesbrasiliensisPb03gi225679132
WGS_ParacoccidioidesbrasiliensisPb18gi226288159
WGS_ParacoccidioidesbrasiliensisPb01gi295658756
Pbra_08192T0
Uree_01212
Cpos_01573
Cimm_RS_v2_01381
Mcan_00507T0
Mgyp_01457T0
Tequ_06848T0
Tton_06329T0
Aben_02936
Tver_00528
Trub_00042T0
Cgra_89544
Clagr2_89544
Xanpa1_60686
Conap1_W9707910T0
Valb_07937T0
Vdah_05512T0
Acral1_1076860
0.3

B. HYP2

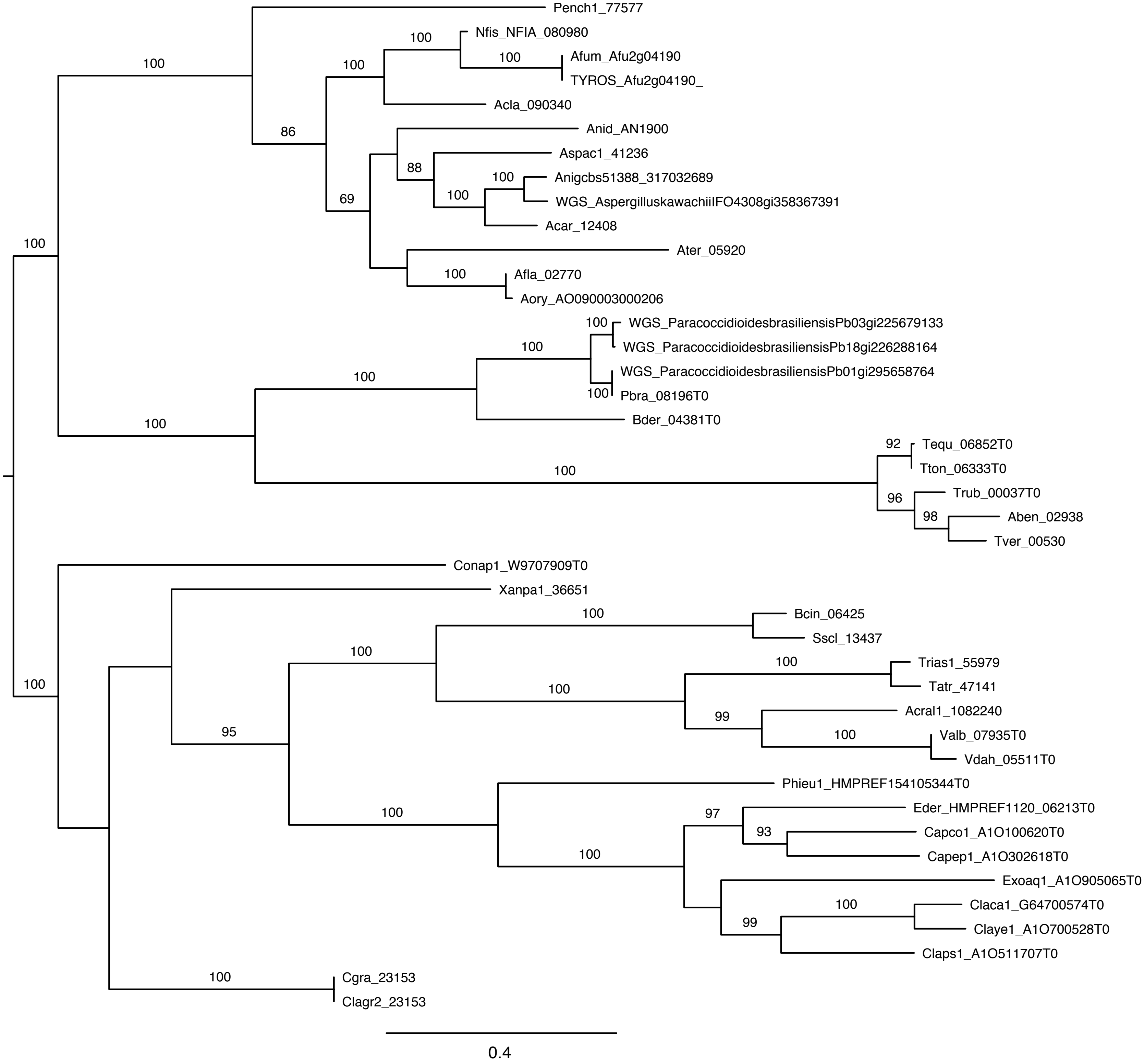
Pench1_77577
Nfis_NFIA_080980
Afum_Afu2g04190
TYROS_Afu2g04190_
Acla_090340
Anid_AN1900
Aspac1_41236
Anigcbs51388_317032689
WGS_AspergilluskawachiiIFO4308gi358367391
Acar_12408
Ater_05920
Afla_02770
Aory_AO090003000206
WGS_ParacoccidioidesbrasiliensisPb03gi225679133
WGS_ParacoccidioidesbrasiliensisPb18gi226288164
WGS_ParacoccidioidesbrasiliensisPb01gi295658764
Pbra_08196T0
Bder_04381T0
Tequ_06852T0
Tton_06333T0
Trub_00037T0
Aben_02938
Tver_00530
Conap1_W9707909T0
Xanpa1_36651
Bcin_06425
Sscl_13437
Trias1_55979
Tatr_47141
Acral1_1082240
Valb_07935T0
Vdah_05511T0
Phieu1_HMPREF154105344T0
Eder_HMPREF1120_06213T0
Capco1_A1O100620T0
Capep1_A1O302618T0
Exoaq1_A1O905065T0
Claca1_G64700574T0
Claye1_A1O700528T0
Claps1_A1O511707T0
Cgra_23153
Clagr2_23153
0.4

C. HPPD

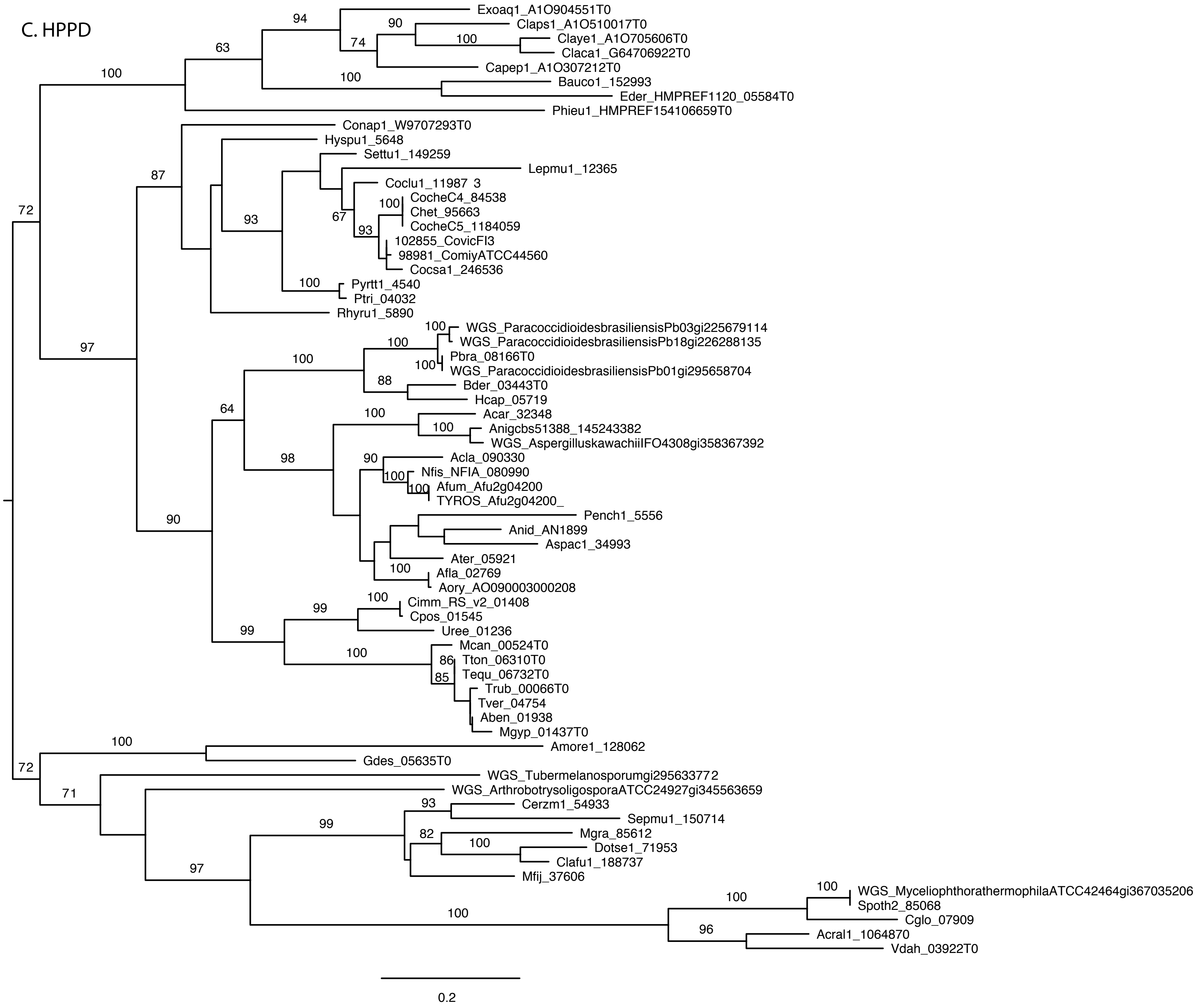
Exoaq1_A1O904551T0
Claps1_A1O510017T0
Claye1_A1O705606T0
Claca1_G64706922T0
Capep1_A1O307212T0
Bauco1_152993
Eder_HMPREF1120_05584T0
Phieu1_HMPREF154106659T0
Conap1_W9707293T0
Hyspu1_5648
Settu1_149259
Lepmu1_12365
Coclu1_11987 3
CocheC4_84538
Chet_95663
CocheC5_1184059
102855_CovicFI3
98981_ComiyATCC44560
Cocsa1_246536
Pyrtt1_4540
Ptri_04032
Rhyru1_5890
WGS_ParacoccidioidesbrasiliensisPb03gi225679114
WGS_ParacoccidioidesbrasiliensisPb18gi226288135
Pbra_08166T0
WGS_ParacoccidioidesbrasiliensisPb01gi295658704
Bder_03443T0
Hcap_05719
Acar_32348
Anigcbs51388_145243382
WGS_AspergilluskawachiiIFO4308gi358367392
Acla_090330
Nfis_NFIA_080990
Afum_Afu2g04200
TYROS_Afu2g04200_
Pench1_5556
Anid_AN1899
Aspac1_34993
Ater_05921
Afla_02769
Aory_AO090003000208
Cimm_RS_v2_01408
Cpos_01545
Uree_01236
Mcan_00524T0
Tton_06310T0
Tequ_06732T0
Trub_00066T0
Tver_04754
Aben_01938
Mgyp_01437T0
Amore1_128062
Gdes_05635T0
WGS_Tubermelanosporumgi295633772
WGS_ArthrobotrysoligosporaATCC24927gi345563659
Cerzm1_54933
Sepmu1_150714
Mgra_85612
Dotse1_71953
Clafu1_188737
Mfij_37606
WGS_MyceliophthorathermophilaATCC42464gi367035206
Spoth2_85068
Cglo_07909
Acral1_1064870
Vdah_03922T0
0.2

D. HMGX

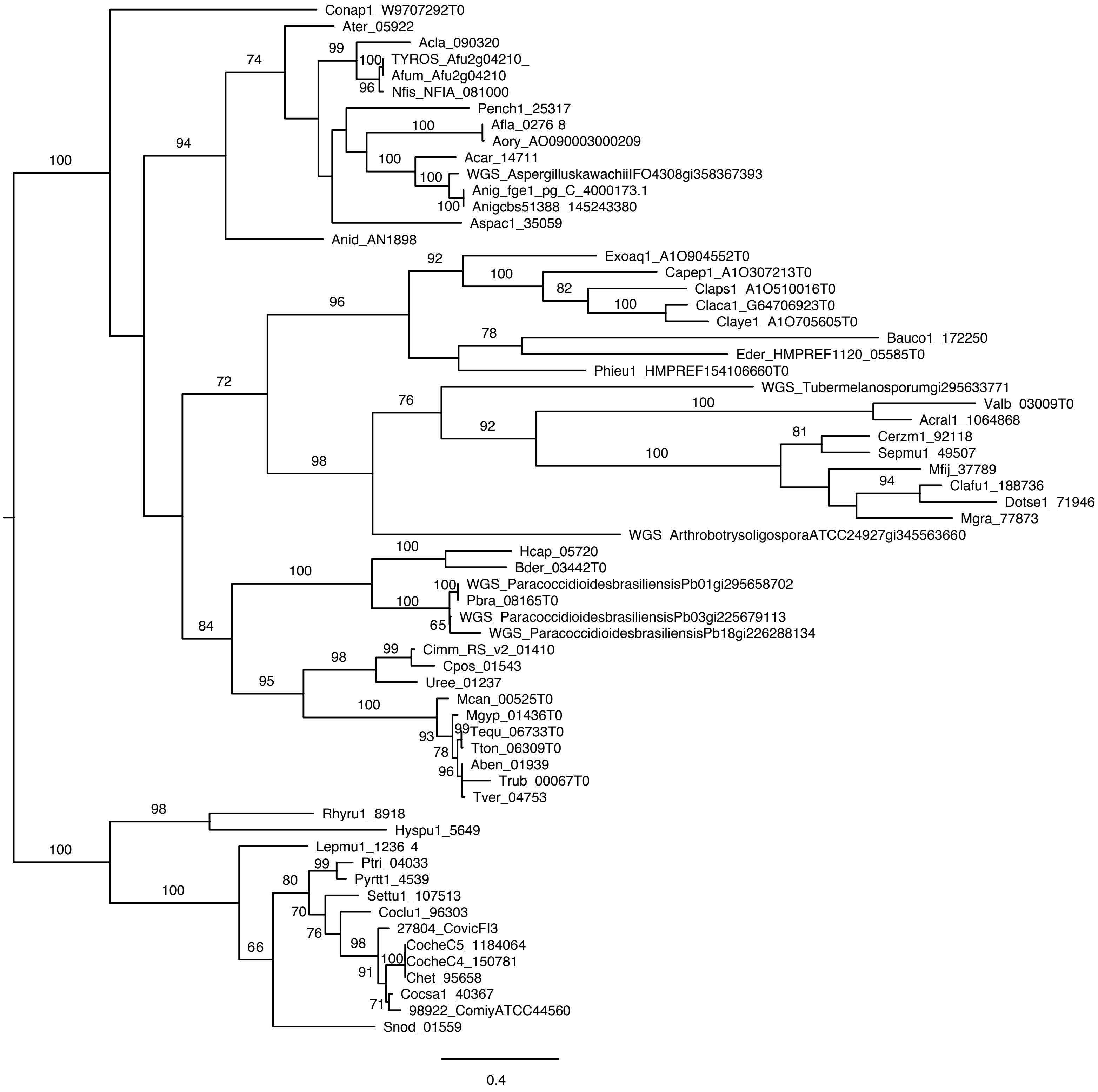

Conap1_W9707292T0
Ater_05922
Acla_090320
TYROS_Afu2g04210_
Afum_Afu2g04210
Nfis_NFIA_081000
Pench1_25317
Afla_0276 8
Aory_AO090003000209
Acar_14711
WGS_AspergilluskawachiiIFO4308gi358367393
Anig_fge1_pg_C_4000173.1
Anigcbs51388_145243380
Aspac1_35059
Anid_AN1898
Exoaq1_A1O904552T0
Capep1_A1O307213T0
Claps1_A1O510016T0
Claca1_G64706923T0
Claye1_A1O705605T0
Bauco1_172250
Eder_HMPREF1120_05585T0
Phieu1_HMPREF154106660T0
WGS_Tubermelanosporumgi295633771
Valb_03009T0
Acral1_1064868
Cerzm1_92118
Sepmu1_49507
Mfij_37789
Clafu1_188736
Dotse1_71946
Mgra_77873
WGS_ArthrobotrysoligosporaATCC24927gi345563660
Hcap_05720
Bder_03442T0
WGS_ParacoccidioidesbrasiliensisPb01gi295658702
Pbra_08165T0
WGS_ParacoccidioidesbrasiliensisPb03gi225679113
WGS_ParacoccidioidesbrasiliensisPb18gi226288134
Cimm_RS_v2_01410
Cpos_01543
Uree_01237
Mcan_00525T0
Mgyp_01436T0
Tequ_06733T0
Tton_06309T0
Aben_01939
Trub_00067T0
Tver_04753
Rhyru1_8918
Hyspu1_5649
Lepmu1_1236 4
Ptri_04033
Pyrtt1_4539
Settu1_107513
Coclu1_96303
27804_CovicFl3
CocheC5_1184064
CocheC4_150781
Chet_95658
Cocsa1_40367
98922_ComiyATCC44560
Snod_01559
0.4

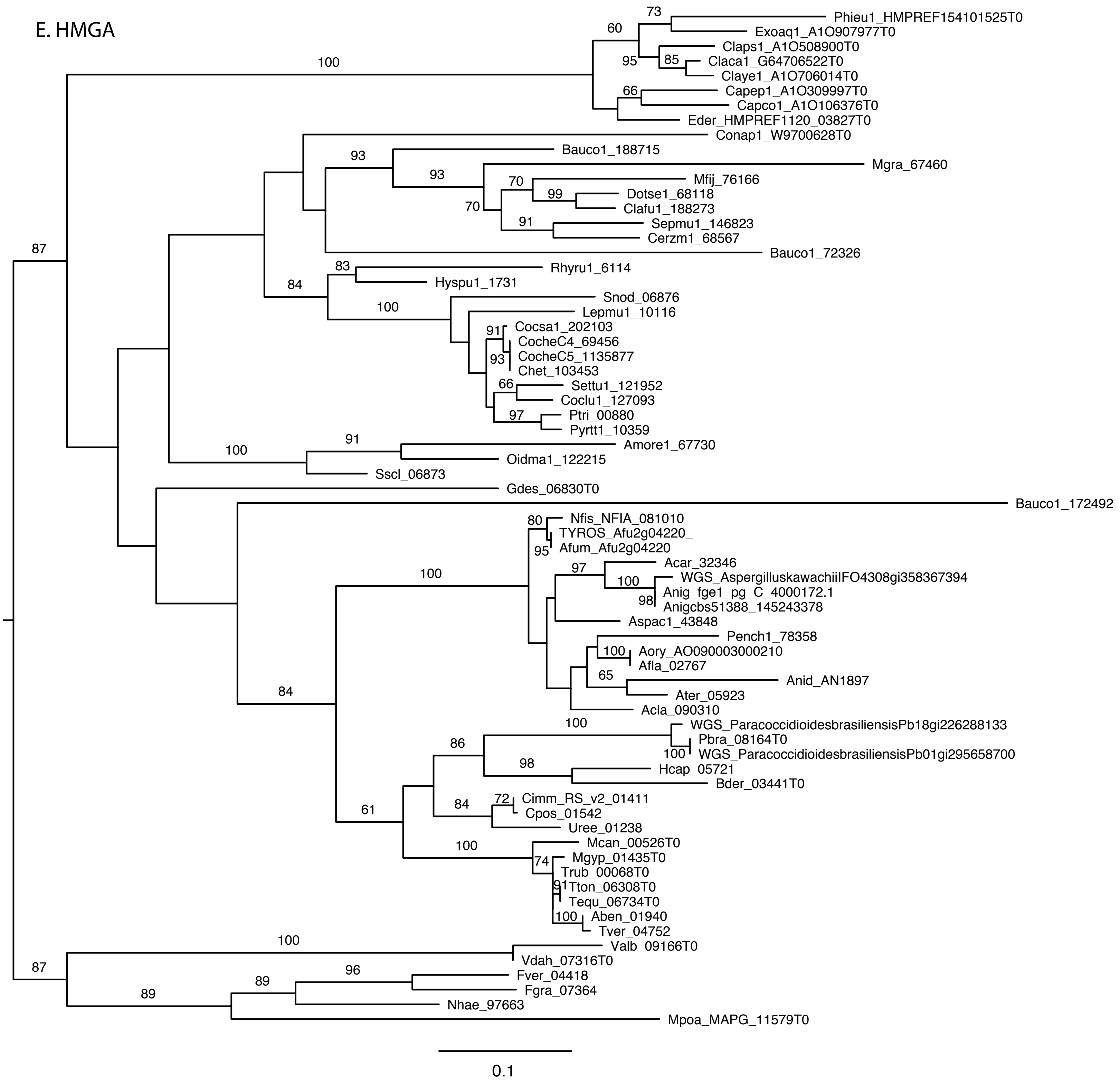

E. HMGA
Phieu1_HMPREF154101525T0
Exoaq1_A1O907977T0
Claps1_A1O508900T0
Claca1_G64706522T0
Claye1_A1O706014T0
Capep1_A1O309997T0
Capco1_A1O106376T0
Eder_HMPREF1120_03827T0
Conap1_W9700628T0
Bauco1_188715
Mgra_67460
Mfij_76166
Dotse1_68118
Clafu1_188273
Sepmu1_146823
Cerzm1_68567
Bauco1_72326
Rhyru1_6114
Hyspu1_1731
Snod_06876
Lepmu1_10116
Cocsa1_202103
CocheC4_69456
CocheC5_1135877
Chet_103453
Settu1_121952
Coclu1_127093
Ptri_00880
Pyrtt1_10359
Amore1_67730
Oidma1_122215
Sscl_06873
Gdes_06830T0
Bauco1_172492
Nfis_NFIA_081010
TYROS_Afu2g04220_
Afum_Afu2g04220
Acar_32346
WGS_AspergilluskawachiiIFO4308gi358367394
Anig_fge1_pg_C_4000172.1
Anigcbs51388_145243378
Aspac1_43848
Pench1_78358
Aory_AO090003000210
Afla_02767
Anid_AN1897
Ater_05923
Acla_090310
WGS_ParacoccidioidesbrasiliensisPb18gi226288133
Pbra_08164T0
WGS_ParacoccidioidesbrasiliensisPb01gi295658700
Hcap_05721
Bder_03441T0
Cimm_RS_v2_01411
Cpos_01542
Uree_01238
Mcan_00526T0
Mgyp_01435T0
Trub_00068T0
Tton_06308T0
Tequ_06734T0
Aben_01940
Tver_04752
Valb_09166T0
Vdah_07316T0
Fver_04418
Fgra_07364
Nhae_97663
Mpoa_MAPG_11579T0
0.1

F. FAHA

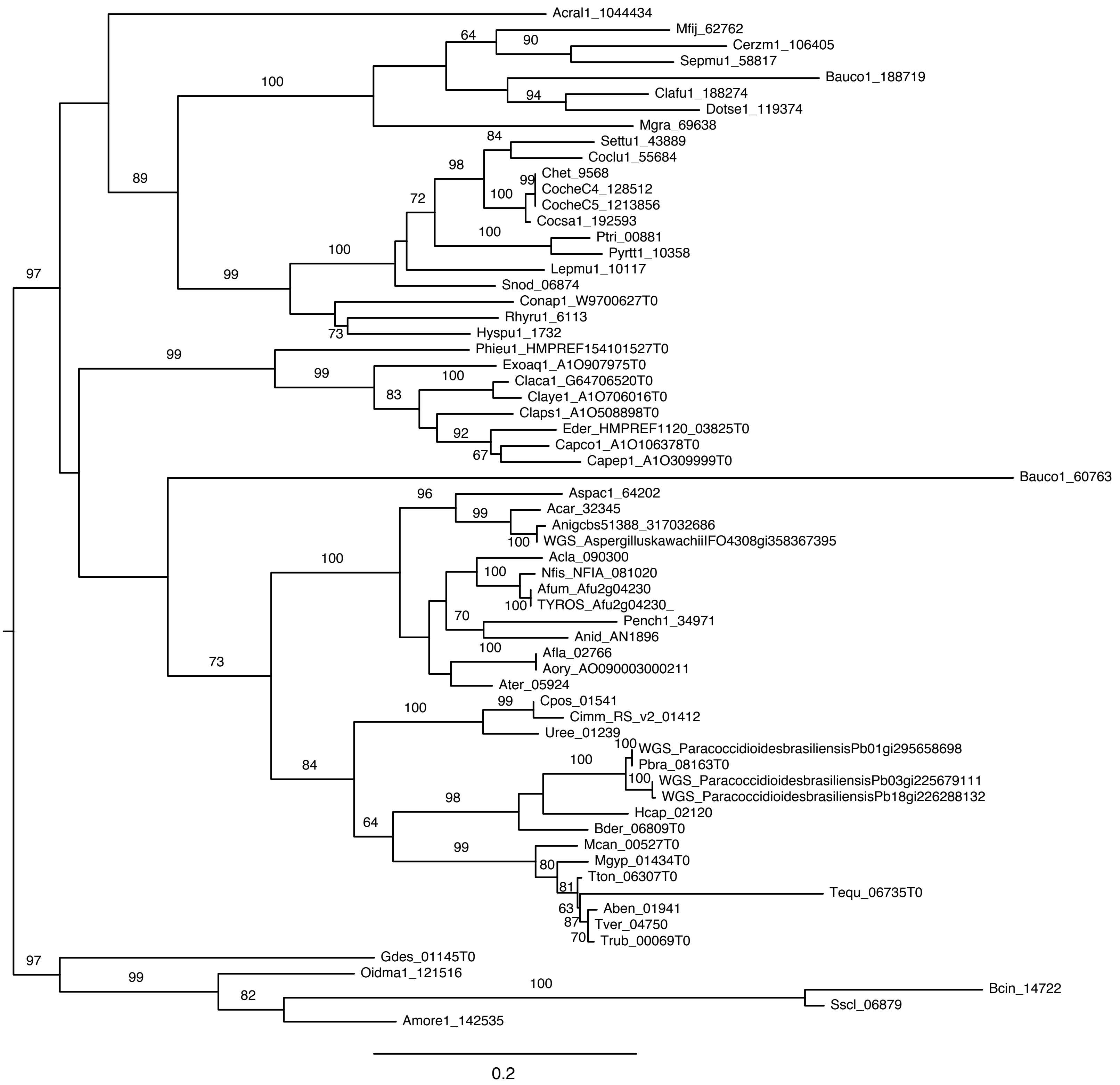

Acral1_1044434
Mfij_62762
Cerzm1_106405
Sepmu1_58817
Bauco1_188719
Clafu1_188274
Dotse1_119374
Mgra_69638
Settu1_43889
Coclu1_55684
Chet_9568
CocheC4_128512
CocheC5_1213856
Cocsa1_192593
Ptri_00881
Pyrtt1_10358
Lepmu1_10117
Snod_06874
Conap1_W9700627T0
Rhyru1_6113
Hyspu1_1732
Phieu1_HMPREF154101527T0
Exoaq1_A1O907975T0
Claca1_G64706520T0
Claye1_A1O706016T0
Claps1_A1O508898T0
Eder_HMPREF1120_03825T0
Capco1_A1O106378T0
Capep1_A1O309999T0
Bauco1_60763
Aspac1_64202
Acar_32345
Anigcbs51388_317032686
WGS_AspergilluskawachiiIFO4308gi358367395
Acla_090300
Nfis_NFIA_081020
Afum_Afu2g04230
TYROS_Afu2g04230_
Pench1_34971
Anid_AN1896
Afla_02766
Aory_AO090003000211
Ater_05924
Cpos_01541
Cimm_RS_v2_01412
Uree_01239
WGS_ParacoccidioidesbrasiliensisPb01gi295658698
Pbra_08163T0
WGS_ParacoccidioidesbrasiliensisPb03gi225679111
WGS_ParacoccidioidesbrasiliensisPb18gi226288132
Hcap_02120
Bder_06809T0
Mcan_00527T0
Mgyp_01434T0
Tton_06307T0
Tequ_06735T0
Aben_01941
Tver_04750
Trub_00069T0
Gdes_01145T0
Oidma1_121516
Bcin_14722
Sscl_06879
Amore1_142535
0.2

G. HMGR

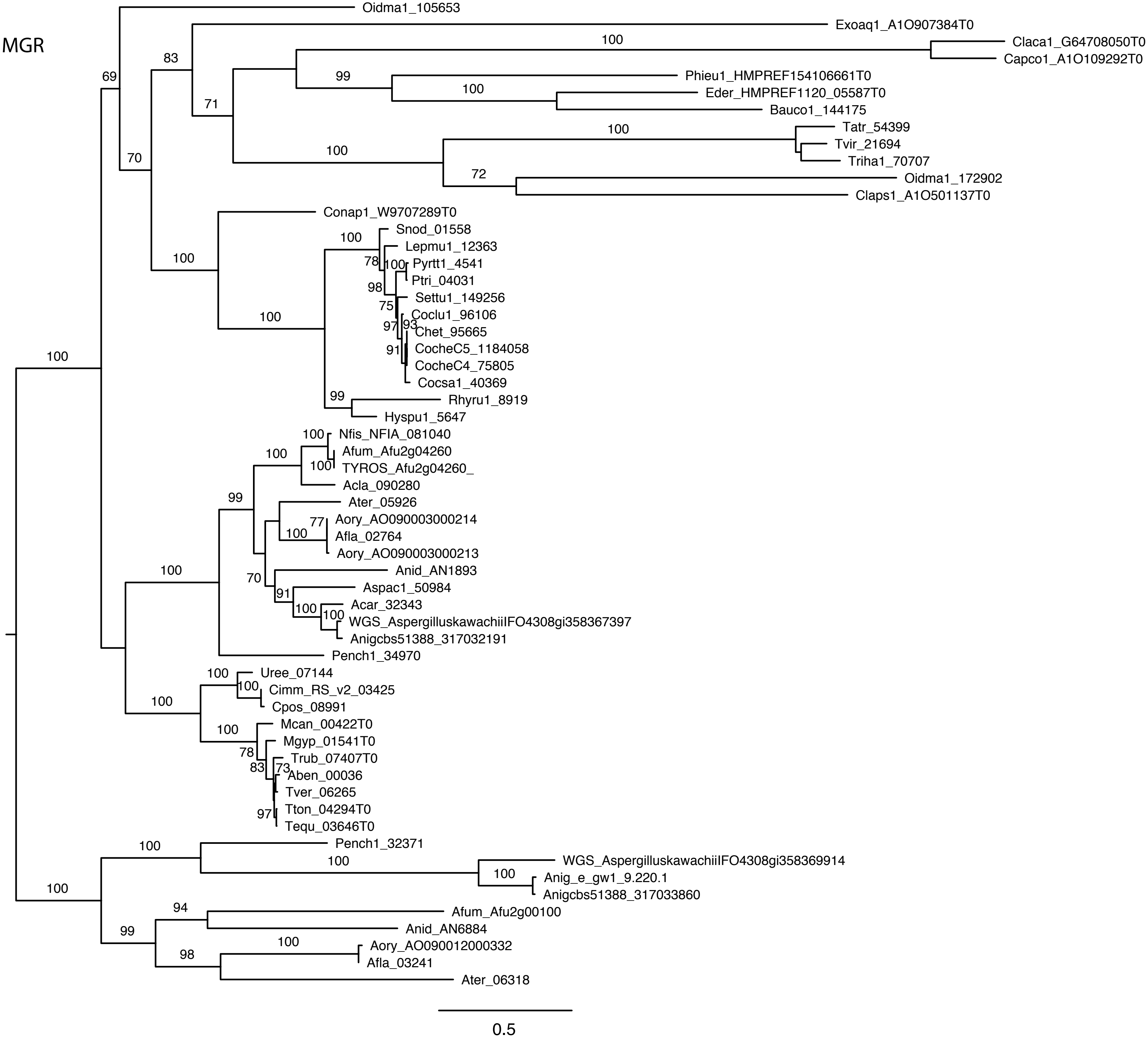
Oidma1_105653
Exoaq1_A1O907384T0
Claca1_G64708050T0
Capco1_A1O109292T0
Phieu1_HMPREF154106661T0
Eder_HMPREF1120_05587T0
Bauco1_144175
Tatr_54399
Tvir_21694
Triha1_70707
Oidma1_172902
Claps1_A1O501137T0
Conap1_W9707289T0
Snod_01558
Lepmu1_12363
Pyrtt1_4541
Ptri_04031
Settu1_149256
Coclu1_96106
Chet_95665
CocheC5_1184058
CocheC4_75805
Cocsa1_40369
Rhyru1_8919
Hyspu1_5647
Nfis_NFIA_081040
Afum_Afu2g04260
TYROS_Afu2g04260_
Acla_090280
Ater_05926
Aory_AO090003000214
Afla_02764
Aory_AO090003000213
Anid_AN1893
Aspac1_50984
Acar_32343
WGS_AspergilluskawachiiIFO4308gi358367397
Anigcbs51388_317032191
Pench1_34970
Uree_07144
Cimm_RS_v2_03425
Cpos_08991
Mcan_00422T0
Mgyp_01541T0
Trub_07407T0
Aben_00036
Tver_06265
Tton_04294T0
Tequ_03646T0
Pench1_32371
WGS_AspergilluskawachiiIFO4308gi358369914
Anig_e_gw1_9.220.1
Anigcbs51388_317033860
Afum_Afu2g00100
Anid_AN6884
Aory_AO090012000332
Afla_03241
Ater_06318
0.5

H. MAIA

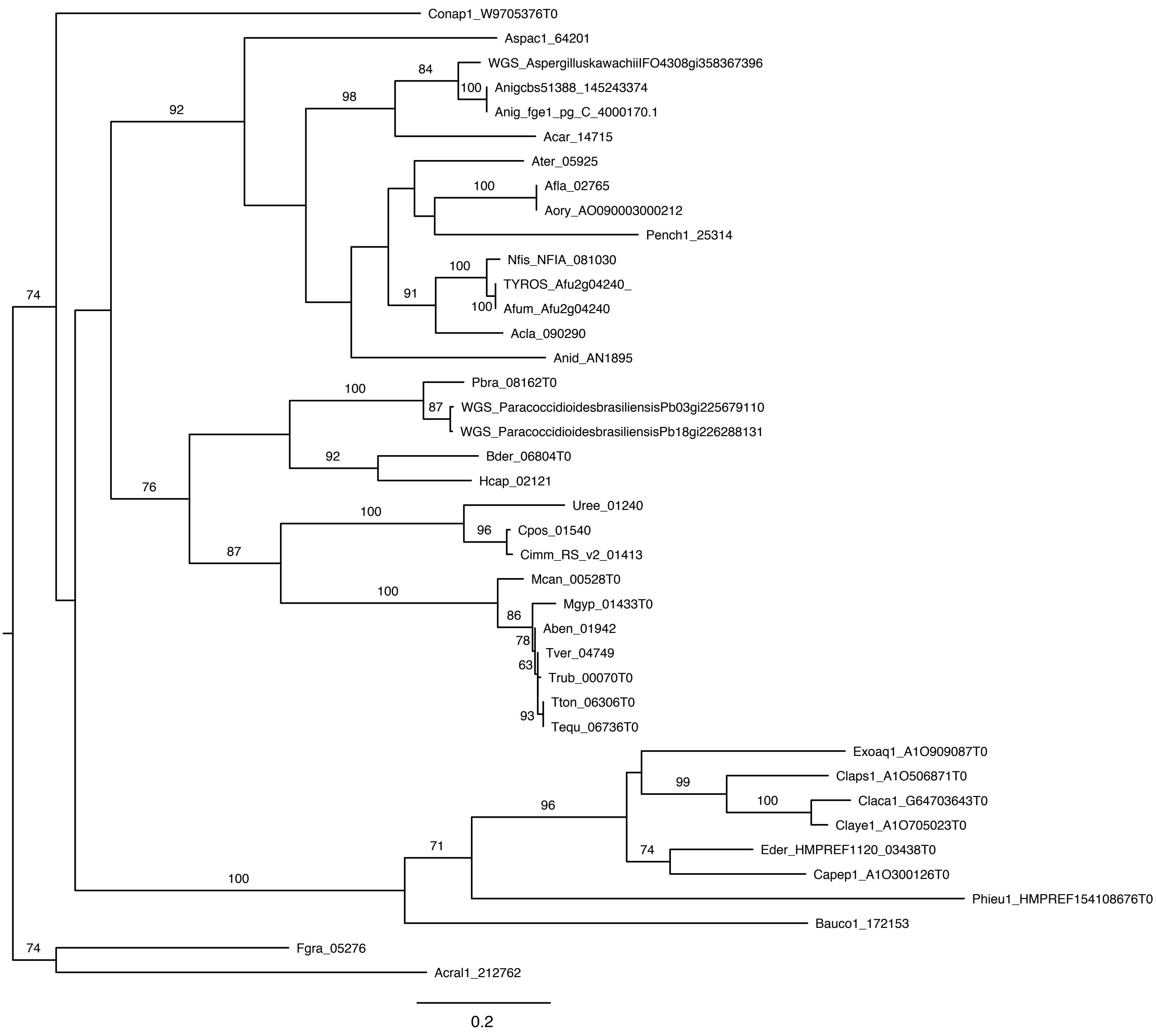

Conap1_W9705376T0
Aspac1_64201
WGS_AspergilluskawachiiIFO4308gi358367396
Anigcbs51388_145243374
Anig_fge1_pg_C_4000170.1
Acar_14715
Ater_05925
Afla_02765
Aory_AO090003000212
Pench1_25314
Nfis_NFIA_081030
TYROS_Afu2g04240_
Afum_Afu2g04240
Acla_090290
Anid_AN1895
Pbra_08162T0
WGS_ParacoccidioidesbrasiliensisPb03gi225679110
WGS_ParacoccidioidesbrasiliensisPb18gi226288131
Bder_06804T0
Hcap_02121
Uree_01240
Cpos_01540
Cimm_RS_v2_01413
Mcan_00528T0
Mgyp_01433T0
Aben_01942
Tver_04749
Trub_00070T0
Tton_06306T0
Tequ_06736T0
Exoaq1_A1O909087T0
Claps1_A1O506871T0
Claca1_G64703643T0
Claye1_A1O705023T0
Eder_HMPREF1120_03438T0
Capep1_A1O300126T0
Phieu1_HMPREF154108676T0
Bauco1_172153
Fgra_05276
Acral1_212762
84
100
98
92
100
100
91
100
74
100
87
92
76
100
96
87
100
86
78
63
93
96
99
100
74
71
100
74
0.2

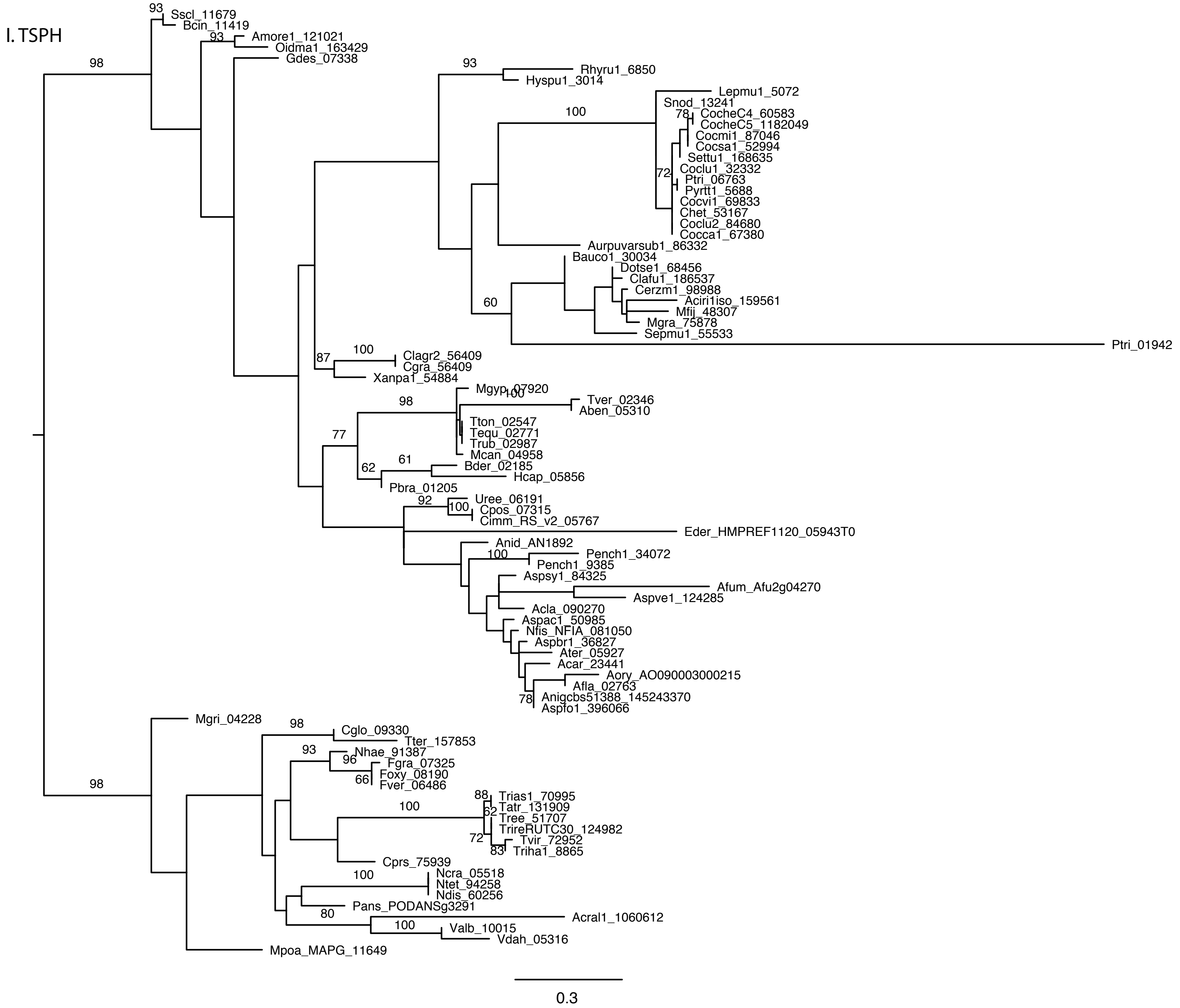

I. TSPH
Sscl_11679
Bcin_11419
Amore1_121021
Oidma1_163429
Gdes_07338
Rhyru1_6850
Hyspu1_3014
Lepmu1_5072
Snod_13241
CocheC4_60583
CocheC5_1182049
Cocmi1_87046
Cocsa1_52994
Settu1_168635
Coclu1_32332
Ptri_06763
Pyrtt1_5688
Cocvi1_69833
Chet_53167
Coclu2_84680
Cocca1_67380
Aurpuvarsub1_86332
Bauco1_30034
Dotse1_68456
Clafu1_186537
Cerzm1_98988
Aciri1iso_159561
Mfij_48307
Mgra_75878
Sepmu1_55533
Ptri_01942
Clagr2_56409
Cgra_56409
Xanpa1_54884
Mgyp_07920
Tver_02346
Aben_05310
Tton_02547
Tequ_02771
Trub_02987
Mcan_04958
Bder_02185
Hcap_05856
Pbra_01205
Uree_06191
Cpos_07315
Cimm_RS_v2_05767
Eder_HMPREF1120_05943T0
Anid_AN1892
Pench1_34072
Pench1_9385
Aspsy1_84325
Afum_Afu2g04270
Aspve1_124285
Acla_090270
Aspac1_50985
Nfis_NFIA_081050
Aspbr1_36827
Ater_05927
Acar_23441
Aory_AO090003000215
Afla_02763
Anigcbs51388_145243370
Aspfo1_396066
Mgri_04228
Cglo_09330
Tter_157853
Nhae_91387
Fgra_07325
Foxy_08190
Fver_06486
Trias1_70995
Tatr_131909
Tree_51707
TrireRUTC30_124982
Tvir_72952
Triha1_8865
Cprs_75939
Ncra_05518
Ntet_94258
Ndis_60256
Pans_PODANSg3291
Acral1_1060612
Valb_10015
Vdah_05316
Mpoa_MAPG_11649
98
93
93
100
78
72
60
100
87
98
100
77
62
61
92
100
100
78
98
98
93
96
66
100
88
62
72
83
100
80
100
0.3

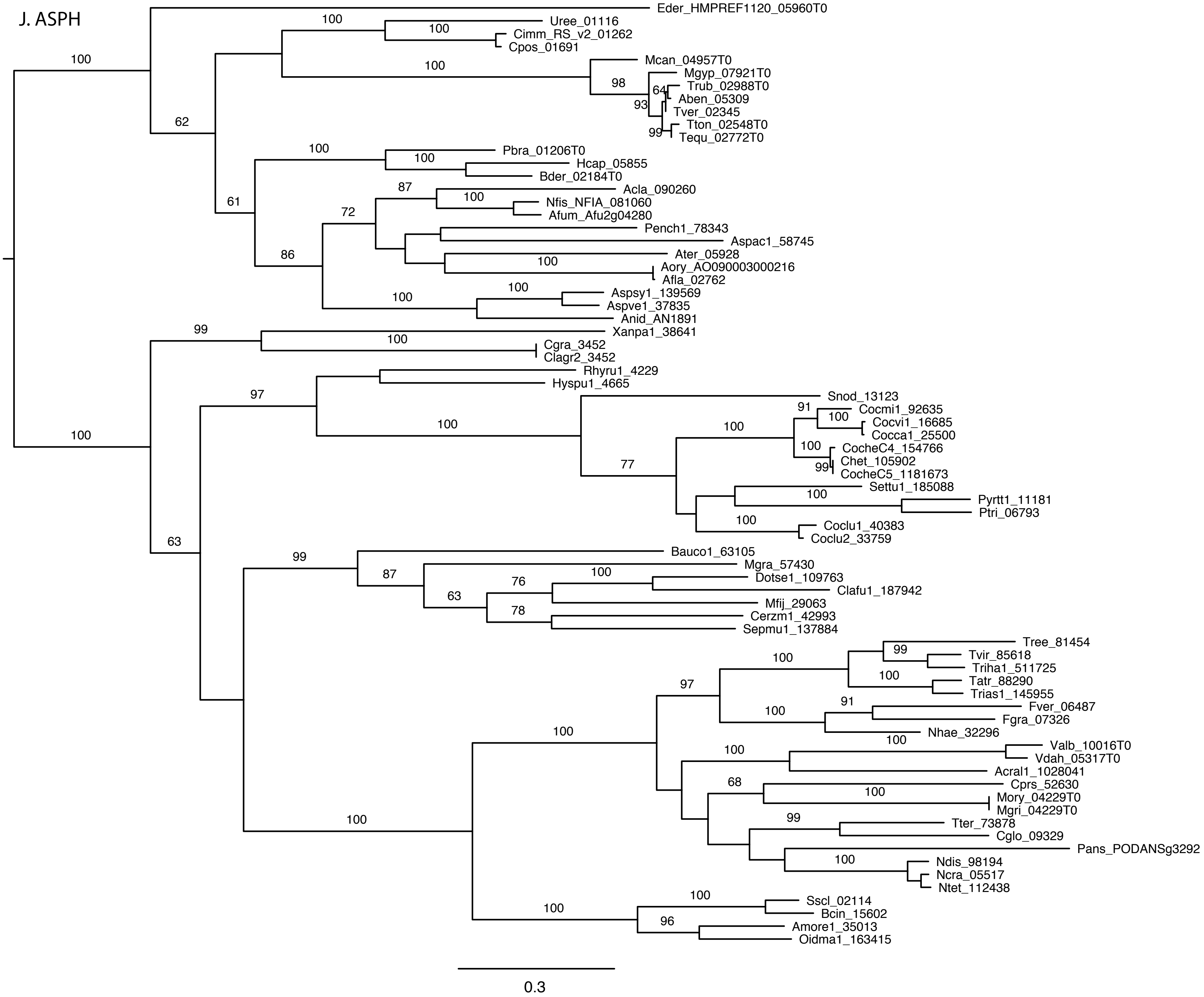
J. ASPH
Eder_HMPREF1120_05960T0
Uree_01116
Cimm_RS_v2_01262
Cpos_01691
Mcan_04957T0
Mgyp_07921T0
Trub_02988T0
Aben_05309
Tver_02345
Tton_02548T0
Tequ_02772T0
Pbra_01206T0
Hcap_05855
Bder_02184T0
Acla_090260
Nfis_NFIA_081060
Afum_Afu2g04280
Pench1_78343
Aspac1_58745
Ater_05928
Aory_AO090003000216
Afla_02762
Aspsy1_139569
Aspve1_37835
Anid_AN1891
Xanpa1_38641
Cgra_3452
Clagr2_3452
Rhyru1_4229
Hyspu1_4665
Snod_13123
Cocmi1_92635
Cocvi1_16685
Cocca1_25500
CocheC4_154766
Chet_105902
CocheC5_1181673
Settu1_185088
Pyrtt1_11181
Ptri_06793
Coclu1_40383
Coclu2_33759
Bauco1_63105
Mgra_57430
Dotse1_109763
Clafu1_187942
Mfij_29063
Cerzm1_42993
Sepmu1_137884
Tree_81454
Tvir_85618
Triha1_511725
Tatr_88290
Trias1_145955
Fver_06487
Fgra_07326
Nhae_32296
Valb_10016T0
Vdah_05317T0
Acral1_1028041
Cprs_52630
Mory_04229T0
Mgri_04229T0
Tter_73878
Cglo_09329
Pans_PODANSg3292
Ndis_98194
Ncra_05517
Ntet_112438
Sscl_02114
Bcin_15602
Amore1_35013
Oidma1_163415
0.3

K. MFSG

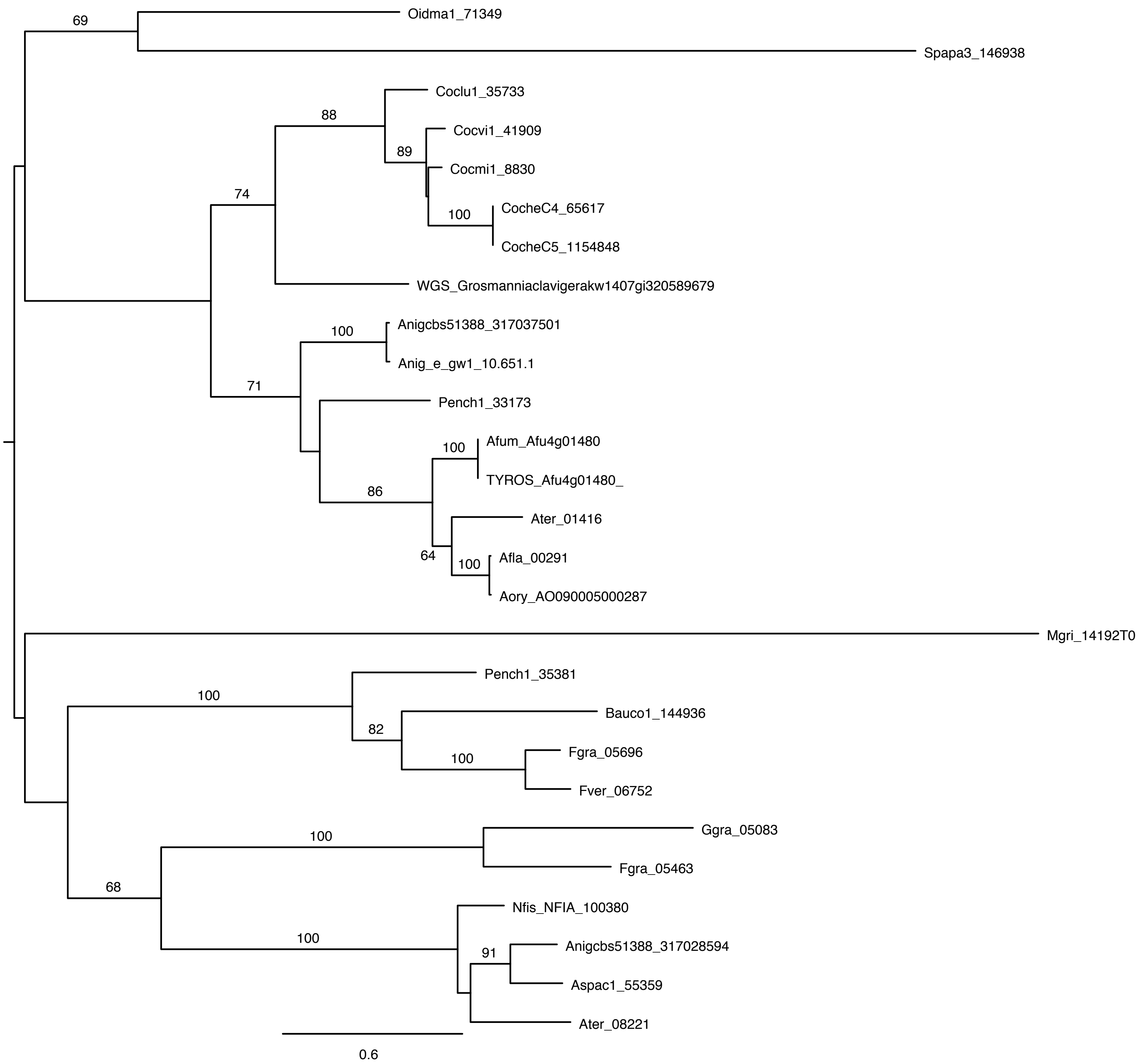

Oidma1_71349
Spapa3_146938
Coclu1_35733
Cocvi1_41909
Cocmi1_8830
CocheC4_65617
CocheC5_1154848
WGS_Grosmanniaclavigerakw1407gi320589679
Anigcbs51388_317037501
Anig_e_gw1_10.651.1
Pench1_33173
Afum_Afu4g01480
TYROS_Afu4g01480_
Ater_01416
Afla_00291
Aory_AO090005000287
Mgri_14192T0
Pench1_35381
Bauco1_144936
Fgra_05696
Fver_06752
Ggra_05083
Fgra_05463
Nfis_NFIA_100380
Anigcbs51388_317028594
Aspac1_55359
Ater_08221
69
88
89
74
100
100
71
100
86
64
100
100
82
100
100
68
100
91
0.6

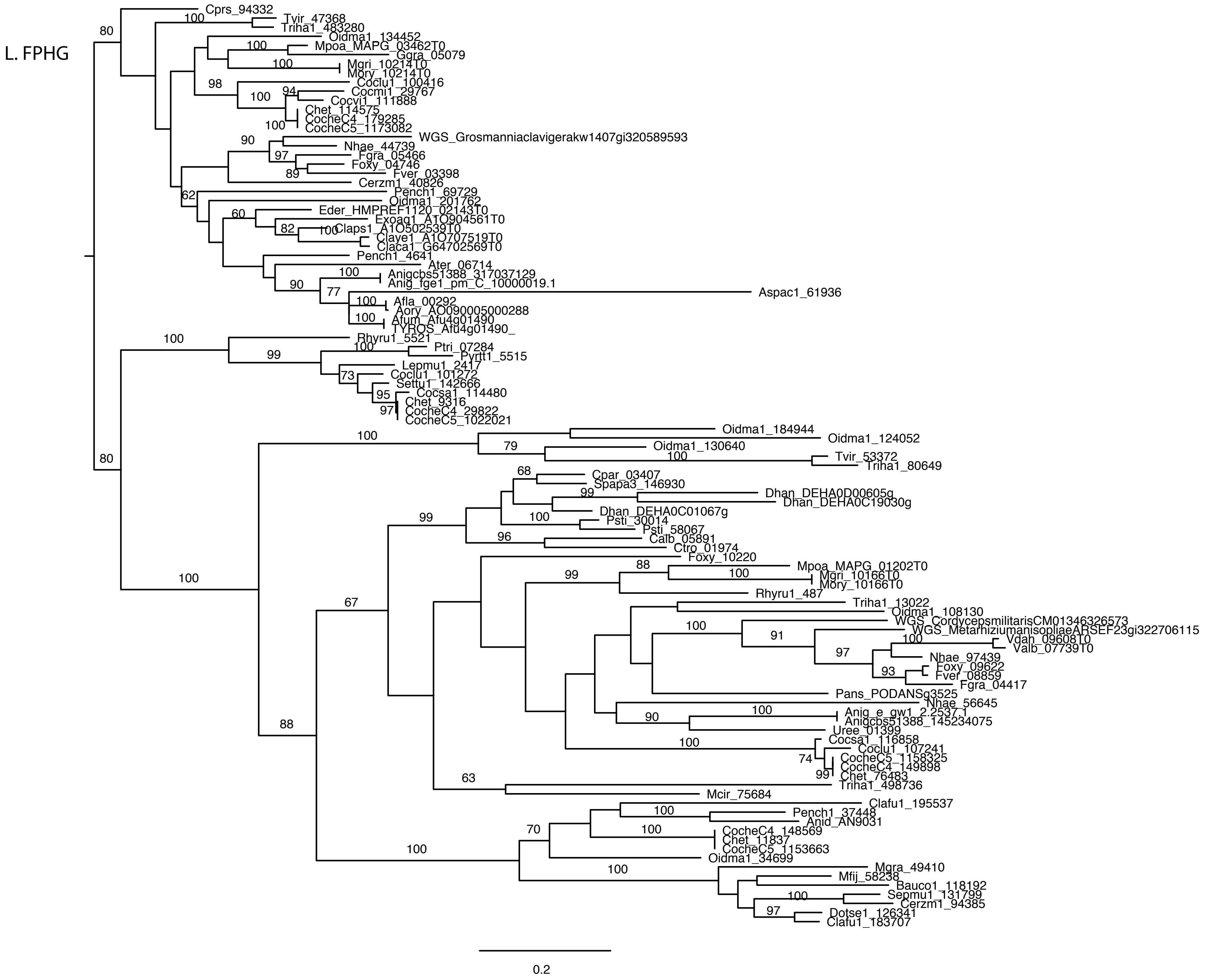
L. FPHG
Cprs_94332
Tvir_47368
Triha1_483280
Oidma1_134452
Mpoa_MAPG_03462T0
Ggra_05079
Mgri_10214T0
Mory_10214T0
Coclu1_100416
Cocmi1_29767
Cocvi1_111888
Chet_114575
CocheC4_179285
CocheC5_1173082
WGS_Grosmanniaclavigerakw1407gi320589593
Nhae_44739
Fgra_05466
Foxy_04746
Fver_03398
Cerzm1_40826
Pench1_69729
Oidma1_201762
Eder_HMPREF1120_02143T0
Exoaq1_A1O904561T0
Claps1_A1O502539T0
Claye1_A1O707519T0
Claca1_G647025697T0
Pench1_4641
Ater_06714
Anigcbs51388_317037129
Anig_fge1_pm_C_10000019.1
Aspac1_61936
Afla_00292
Aory_AO090005000288
Afum_Afu4g01490
TYROS_Afu4g01490_
Rhyru1_5521
Ptri_07284
Pyrtt1_5515
Lepmu1_2417
Coclu1_101272
Settu1_142666
Cocsa1_114480
Chet_9316
CocheC4_29822
CocheC5_1022021
Oidma1_184944
Oidma1_124052
Oidma1_130640
Tvir_53372
Triha1_80649
Cpar_03407
Spapa3_146930
Dhan_DEHA0D00605g
Dhan_DEHA0C19030g
Dhan_DEHA0C01067g
Psti_30014
Psti_58067
Calb_05891
Ctro_01974
Foxy_10220
Mpoa_MAPG_01202T0
Mgri_10166T0
Mory_10166T0
Rhyru1_487
Triha1_13022
Oidma1_108130
WGS_CordycepsmilitarisCM01346326573
WGS_MetarhiziumanisopliaeARSEF23gi322706115
Vdah_09608T0
Valb_07739T0
Nhae_97439
Foxy_09622
Fver_08859
Fgra_04417
Pans_PODANSg3525
Nhae_56645
Anig_e_gw1_2.2537_1
Anigcbs51388_145234075
Uree_01399
Cocsa1_116858
Coclu1_107241
CocheC5_1158325
CocheC4_149898
Chet_76483
Triha1_498736
Mcir_75684
Clafu1_195537
Pench1_37448
Anid_AN9031
CocheC4_148569
Chet_11837
CocheC5_1153663
Oidma1_34699
Mgra_49410
Mfij_58238
Bauco1_118192
Sepmu1_131799
Cerzm1_94385
Dotse1_126341
Clafu1_183707
0.2

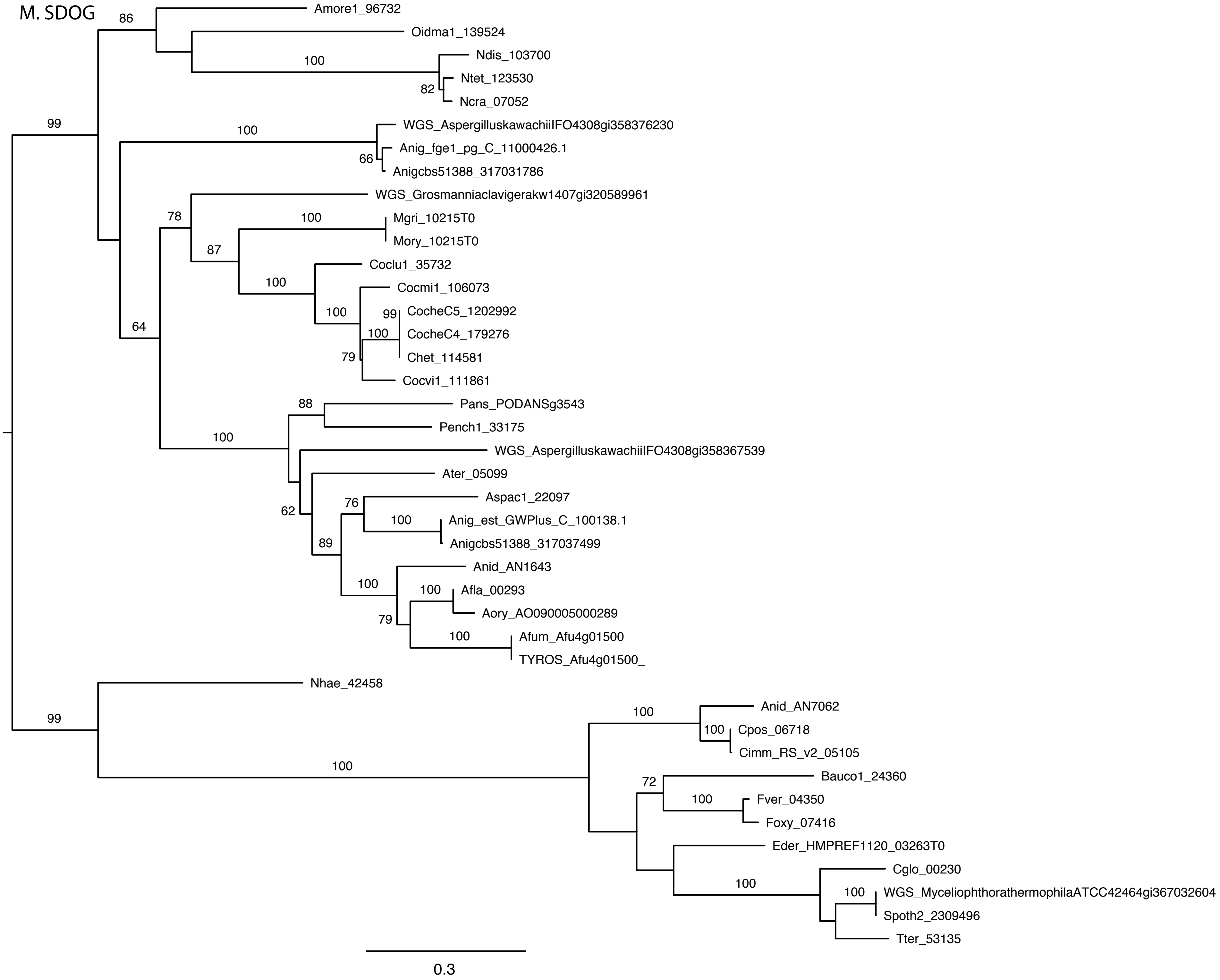

M. SDOG
Amore1_96732
Oidma1_139524
Ndis_103700
Ntet_123530
Ncra_07052
WGS_AspergilluskawachiiIFO4308gi358376230
Anig_fge1_pg_C_11000426.1
Anigcbs51388_317031786
WGS_Grosmanniaclavigerakw1407gi320589961
Mgri_10215T0
Mory_10215T0
Coclu1_35732
Cocmi1_106073
CocheC5_1202992
CocheC4_179276
Chet_114581
Cocvi1_111861
Pans_PODANSg3543
Pench1_33175
WGS_AspergilluskawachiiIFO4308gi358367539
Ater_05099
Aspac1_22097
Anig_est_GWPlus_C_100138.1
Anigcbs51388_317037499
Anid_AN1643
Afla_00293
Aory_AO090005000289
Afum_Afu4g01500
TYROS_Afu4g01500_
Nhae_42458
Anid_AN7062
Cpos_06718
Cimm_RS_v2_05105
Bauco1_24360
Fver_04350
Foxy_07416
Eder_HMPREF1120_03263T0
Cglo_00230
WGS_MyceliophthorathermophilaATCC42464gi367032604
Spoth2_2309496
Tter_53135
0.3

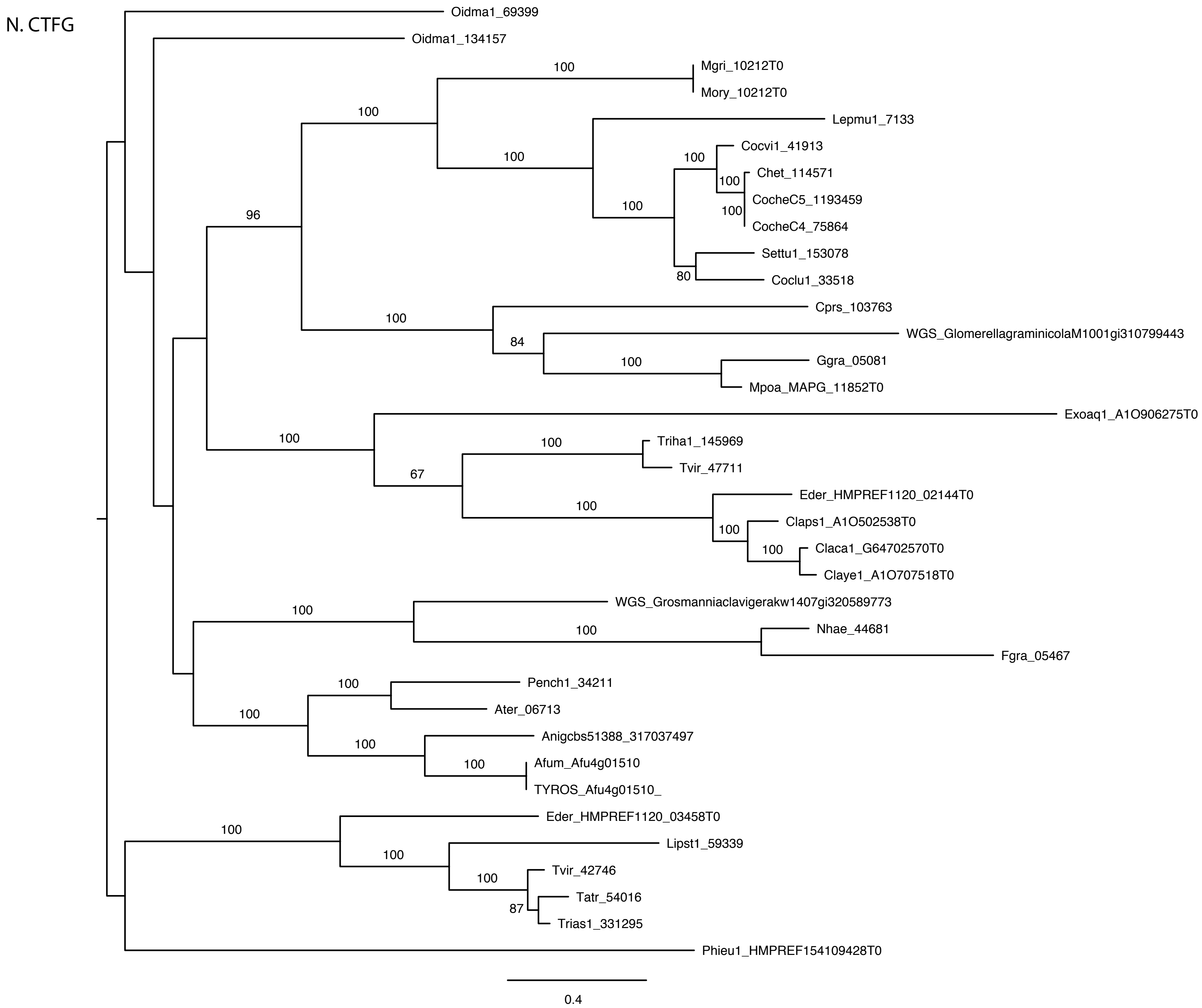

N. CTFG
Oidma1_69399
Oidma1_134157
Mgri_10212T0
Mory_10212T0
Lepmu1_7133
Cocvi1_41913
Chet_114571
CocheC5_1193459
CocheC4_75864
Settu1_153078
Coclu1_33518
Cprs_103763
WGS_GlomerellagraminicolaM1001gi310799443
Ggra_05081
Mpoa_MAPG_11852T0
Exoaq1_A1O906275T0
Triha1_145969
Tvir_47711
Eder_HMPREF1120_02144T0
Claps1_A1O502538T0
Claca1_G64702570T0
Claye1_A1O707518T0
WGS_Grosmanniaclavigerakw1407gi320589773
Nhae_44681
Fgra_05467
Pench1_34211
Ater_06713
Anigcbs51388_317037497
Afum_Afu4g01510
TYROS_Afu4g01510_
Eder_HMPREF1120_03458T0
Lipst1_59339
Tvir_42746
Tatr_54016
Trias1_331295
Phieu1_HMPREF154109428T0
0.4

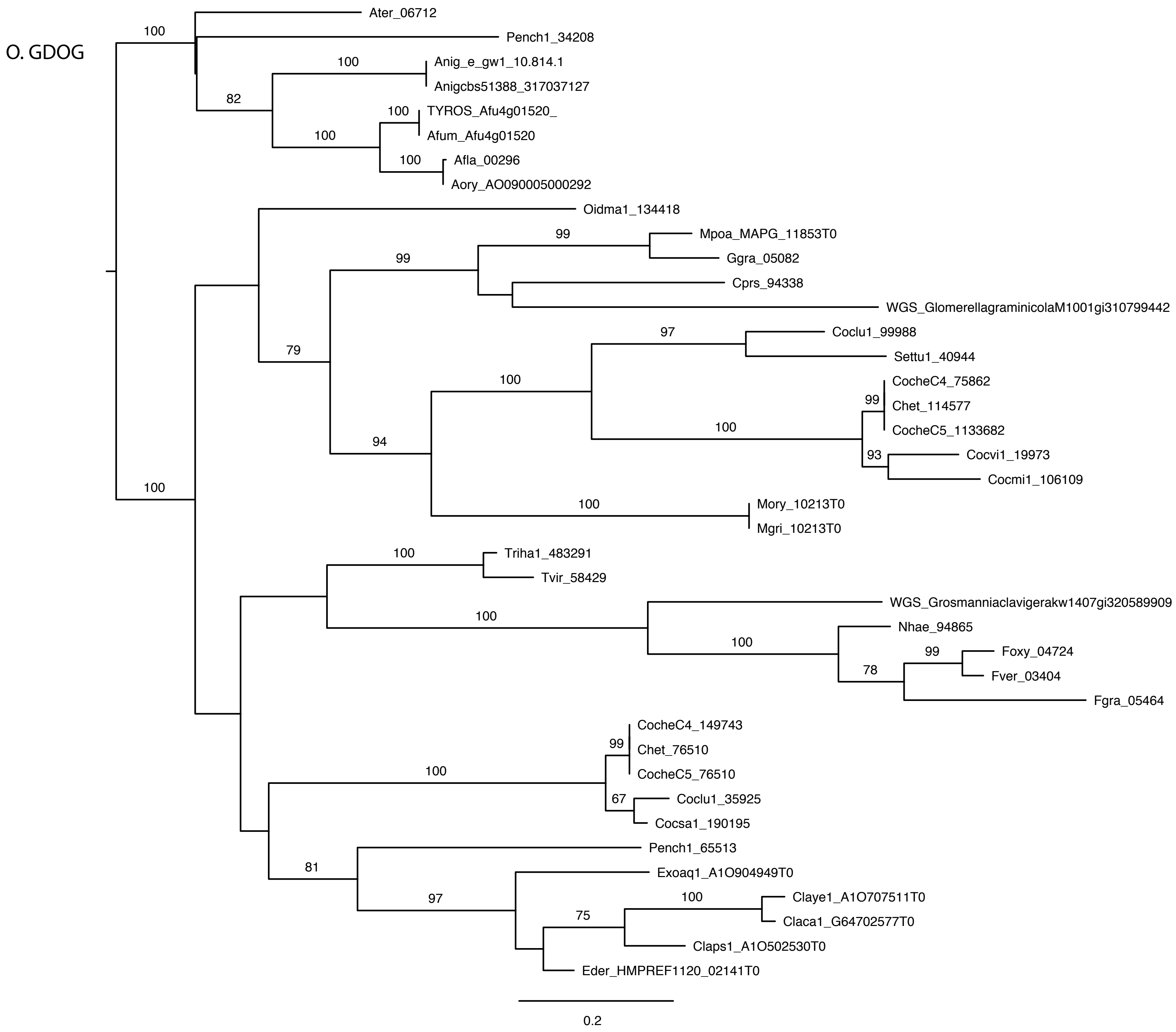

O. GDOG
Ater_06712
Pench1_34208
Anig_e_gw1_10.814.1
Anigcbs51388_317037127
TYROS_Afu4g01520_
Afum_Afu4g01520
Afla_00296
Aory_AO090005000292
Oidma1_134418
Mpoa_MAPG_11853T0
Ggra_05082
Cprs_94338
WGS_GlomerellagraminicolaM1001gi310799442
Coclu1_99988
Settu1_40944
CocheC4_75862
Chet_114577
CocheC5_1133682
Cocvi1_19973
Cocmi1_106109
Mory_10213T0
Mgri_10213T0
Triha1_483291
Tvir_58429
WGS_Grosmanniaclavigerakw1407gi320589909
Nhae_94865
Foxy_04724
Fver_03404
Fgra_05464
CocheC4_149743
Chet_76510
CocheC5_76510
Coclu1_35925
Cocsa1_190195
Pench1_65513
Exoaq1_A1O904949T0
Claye1_A1O707511T0
Claca1_G64702577T0
Claps1_A1O502530T0
Eder_HMPREF1120_02141T0
0.2

P. SAHG

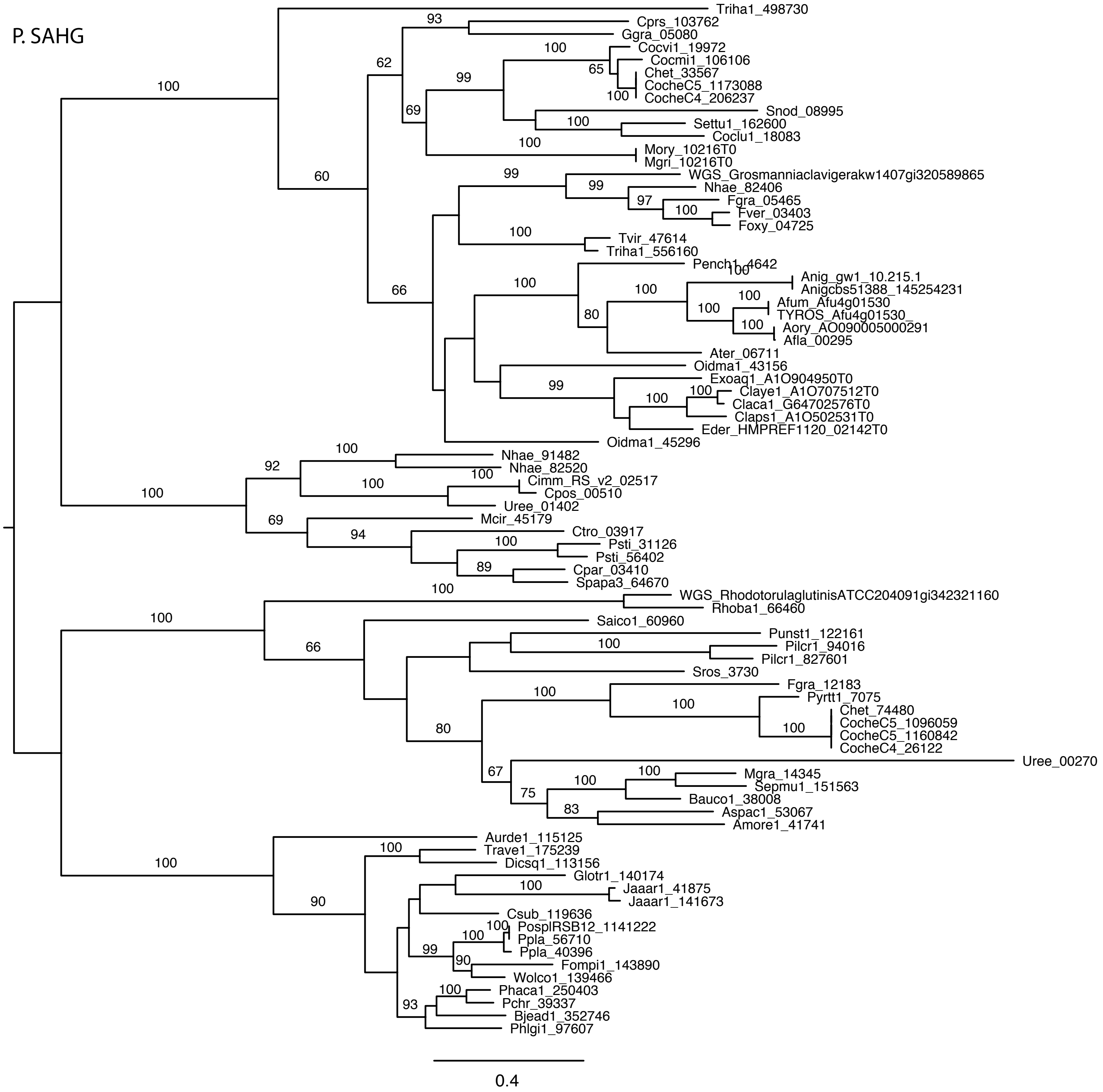

Triha1_498730
Cprs_103762
Ggra_05080
Cocvi1_19972
Cocmi1_106106
Chet_33567
CocheC5_1173088
CocheC4_206237
Snod_08995
Settu1_162600
Coclu1_18083
Mory_10216T0
Mgri_10216T0
WGS_Grosmanniaclavigerakw1407gi320589865
Nhae_82406
Fgra_05465
Fver_03403
Foxy_04725
Tvir_47614
Triha1_556160
Pench1_4642
Anig_gw1_10.215.1
Anigcbs51388_145254231
Afum_Afu4g01530
TYROS_Afu4g01530_
Aory_AO090005000291
Afla_00295
Ater_06711
Oidma1_43156
Exoaq1_A1O904950T0
Claye1_A1O707512T0
Claca1_G64702576T0
Claps1_A1O502531T0
Eder_HMPREF1120_02142T0
Oidma1_45296
Nhae_91482
Nhae_82520
Cimm_RS_v2_02517
Cpos_00510
Uree_01402
Mcir_45179
Ctro_03917
Psti_31126
Psti_56402
Cpar_03410
Spapa3_64670
WGS_RhodotorulaglutinisATCC204091gi342321160
Rhoba1_66460
Saico1_60960
Punst1_122161
Pilcr1_94016
Pilcr1_827601
Sros_3730
Fgra_12183
Pyrtt1_7075
Chet_74480
CocheC5_1096059
CocheC5_1160842
CocheC4_26122
Uree_00270
Mgra_14345
Sepmu1_151563
Bauco1_38008
Aspac1_53067
Amore1_41741
Aurde1_115125
Trave1_175239
Dicsq1_113156
Glotr1_140174
Jaaar1_41875
Jaaar1_141673
Csub_119636
PosplRSB12_1141222
Ppla_56710
Ppla_40396
Fompi1_143890
Wolco1_139466
Phaca1_250403
Pchr_39337
Bjead1_352746
Phlgi1_97607
0.4

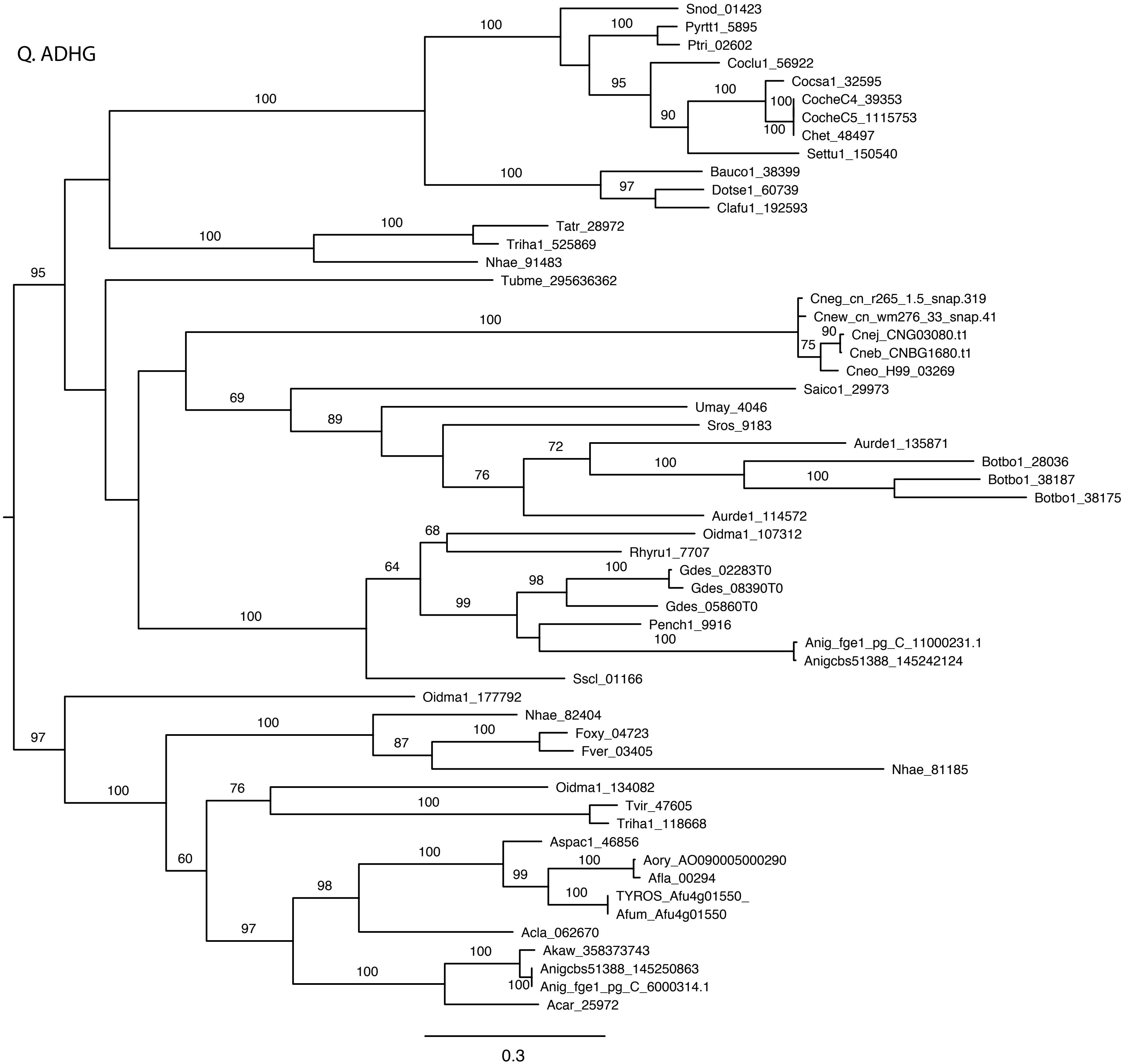
Q. ADHG
Snod_01423
Pyrtt1_5895
Ptri_02602
Coclu1_56922
Cocsa1_32595
CocheC4_39353
CocheC5_1115753
Chet_48497
Settu1_150540
Bauco1_38399
Dotse1_60739
Clafu1_192593
Tatr_28972
Triha1_525869
Nhae_91483
Tubme_295636362
Cneg_cn_r265_1.5_snap.319
Cnew_cn_wm276_33_snap.41
Cnej_CNG03080.t1
Cneb_CNBG1680.t1
Cneo_H99_03269
Saico1_29973
Umay_4046
Sros_9183
Aurde1_135871
Botbo1_28036
Botbo1_38187
Botbo1_38175
Aurde1_114572
Oidma1_107312
Rhyru1_7707
Gdes_02283T0
Gdes_08390T0
Gdes_05860T0
Pench1_9916
Anig_fge1_pg_C_11000231.1
Anigcbs51388_145242124
Sscl_01166
Oidma1_177792
Nhae_82404
Foxy_04723
Fver_03405
Nhae_81185
Oidma1_134082
Tvir_47605
Triha1_118668
Aspac1_46856
Aory_AO090005000290
Afla_00294
TYROS_Afu4g01550_
Afum_Afu4g01550
Acla_062670
Akaw_358373743
Anigcbs51388_145250863
Anig_fge1_pg_C_6000314.1
Acar_25972
0.3

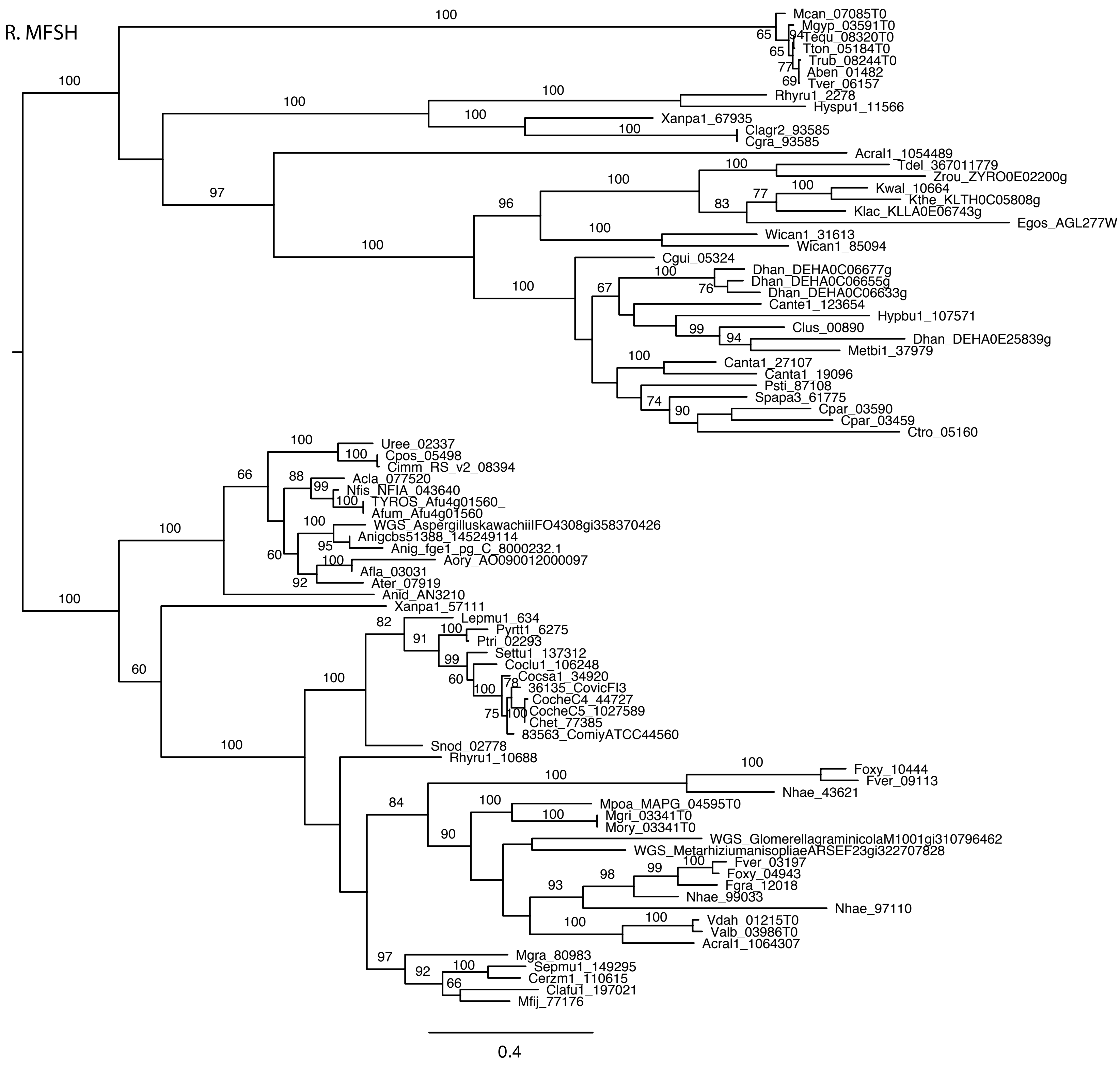
R. MFSH
Mcan_07085T0
Mgyp_03591T0
Tequ_08320T0
Tton_05184T0
Trub_08244T0
Aben_01482
Tver_06157
Rhyru1_2278
Hyspu1_11566
Xanpa1_67935
Clagr2_93585
Cgra_93585
Acral1_1054489
Tdel_367011779
Zrou_ZYRO0E02200g
Kwal_10664
Kthe_KLTH0C05808g
Klac_KLLA0E06743g
Egos_AGL277W
Wican1_31613
Wican1_85094
Cgui_05324
Dhan_DEHA0C06677g
Dhan_DEHA0C06655g
Dhan_DEHA0C06633g
Cante1_123654
Hypbu1_107571
Clus_00890
Dhan_DEHA0E25839g
Metbi1_37979
Canta1_27107
Canta1_19096
Psti_87108
Spapa3_61775
Cpar_03590
Cpar_03459
Ctro_05160
Uree_02337
Cpos_05498
Cimm_RS_v2_08394
Acla_077520
Nfis_NFIA_043640
TYROS_Afu4g01560_
Afum_Afu4g01560
WGS_AspergilluskawachiiIFO4308gi358370426
Anigcbs51388_145249114
Anig_fge1_pg_C_8000232.1
Aory_AO090012000097
Afla_03031
Ater_07919
Anid_AN3210
Xanpa1_57111
Lepmu1_634
Pyrtt1_6275
Ptri_02293
Settu1_137312
Coclu1_106248
Cocsa1_34920
36135_CovicFl3
CocheC4_44727
CocheC5_1027589
Chet_77385
83563_ComiyATCC44560
Snod_02778
Rhyru1_10688
Foxy_10444
Fver_09113
Nhae_43621
Mpoa_MAPG_04595T0
Mgri_03341T0
Mory_03341T0
WGS_GlomerellagraminicolaM1001gi310796462
WGS_MetarhiziumanisopliaeARSEF23gi322707828
Fver_03197
Foxy_04943
Fgra_12018
Nhae_99033
Nhae_97110
Vdah_01215T0
Valb_03986T0
Acral1_1064307
Mgra_80983
Sepmu1_149295
Cerzm1_110615
Clafu1_197021
Mfij_77176
0.4

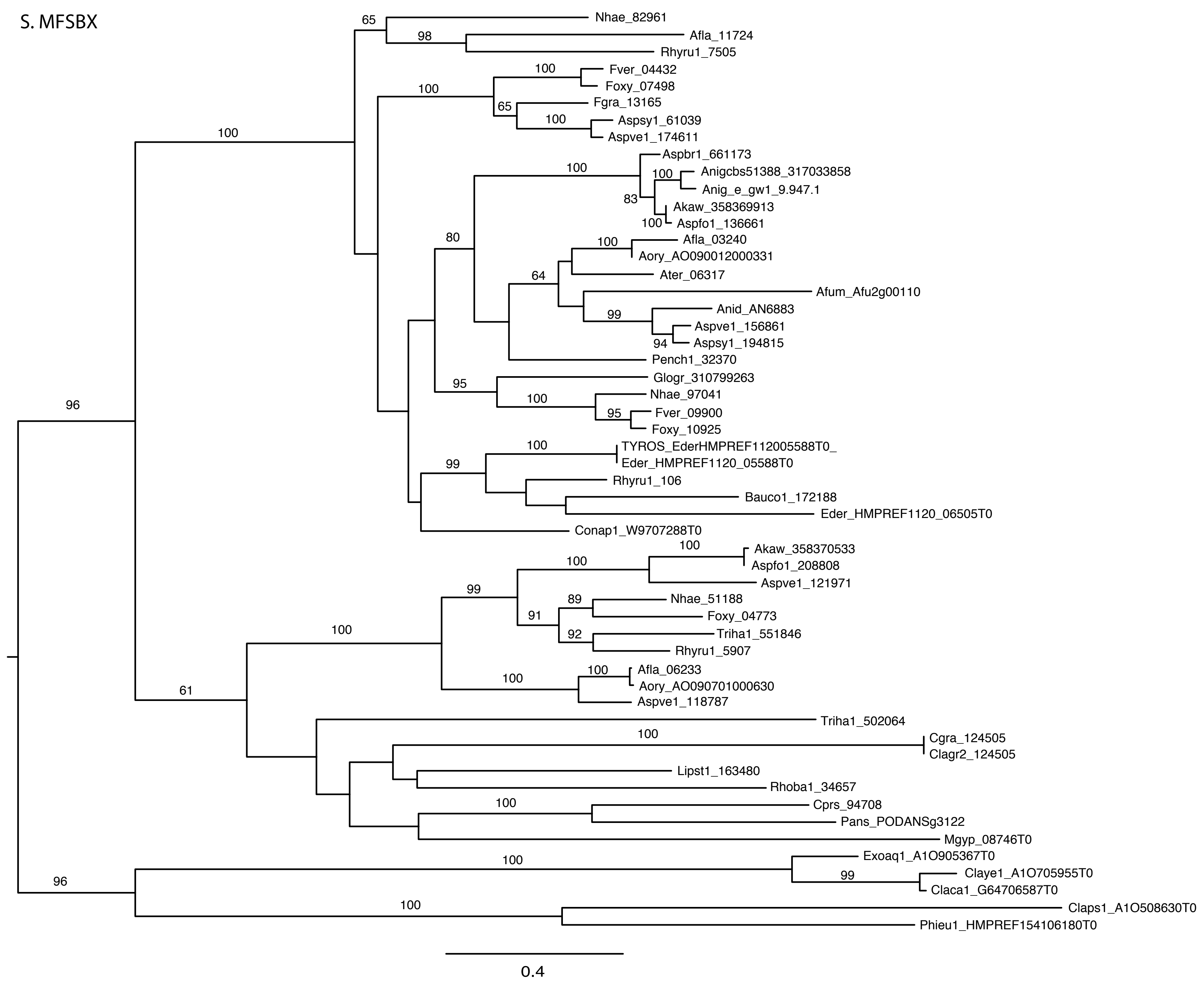
S. MFSBX
Nhae_82961
Afla_11724
Rhyru1_7505
Fver_04432
Foxy_07498
Fgra_13165
Aspsy1_61039
Aspve1_174611
Aspbr1_661173
Anigcbs51388_317033858
Anig_e_gw1_9.947.1
Akaw_358369913
Aspfo1_136661
Afla_03240
Aory_AO090012000331
Ater_06317
Afum_Afu2g00110
Anid_AN6883
Aspve1_156861
Aspsy1_194815
Pench1_32370
Glogr_310799263
Nhae_97041
Fver_09900
Foxy_10925
TYROS_EderHMPREF112005588T0_
Eder_HMPREF1120_05588T0
Rhyru1_106
Bauco1_172188
Eder_HMPREF1120_06505T0
Conap1_W9707288T0
Akaw_358370533
Aspfo1_208808
Aspve1_121971
Nhae_51188
Foxy_04773
Triha1_551846
Rhyru1_5907
Afla_06233
Aory_AO090701000630
Aspve1_118787
Triha1_502064
Cgra_124505
Clagr2_124505
Lipst1_163480
Rhoba1_34657
Cprs_94708
Pans_PODANSg3122
Mgyp_08746T0
Exoaq1_A1O905367T0
Claye1_A1O705955T0
Claca1_G64706587T0
Claps1_A1O508630T0
Phieu1_HMPREF154106180T0
0.4

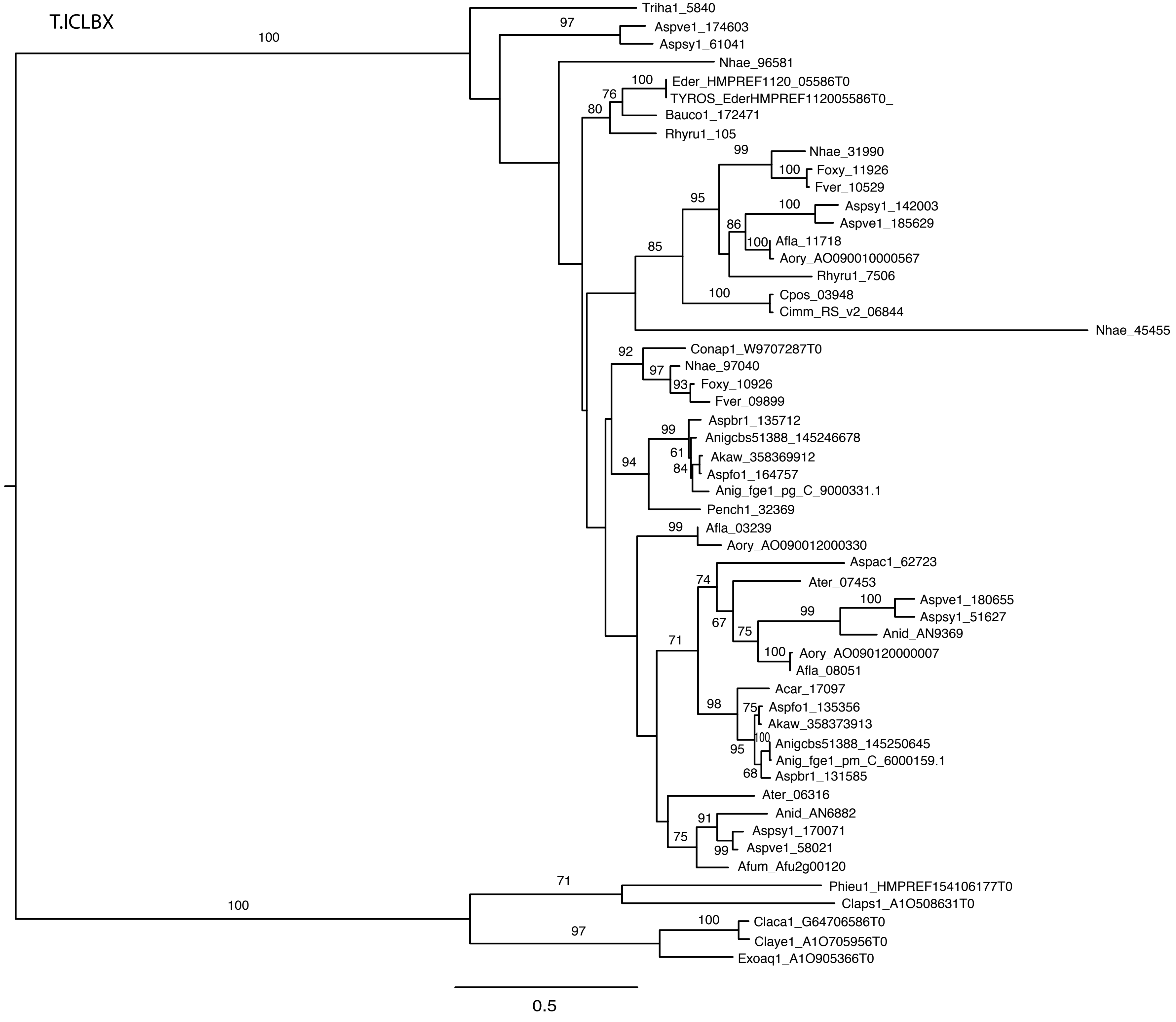
T.ICLBX
Triha1_5840
Aspve1_174603
Aspsy1_61041
Nhae_96581
Eder_HMPREF1120_05586T0
TYROS_EderHMPREF112005586T0_
Bauco1_172471
Rhyru1_105
Nhae_31990
Foxy_11926
Fver_10529
Aspsy1_142003
Aspve1_185629
Afla_11718
Aory_AO090010000567
Rhyru1_7506
Cpos_03948
Cimm_RS_v2_06844
Nhae_45455
Conap1_W9707287T0
Nhae_97040
Foxy_10926
Fver_09899
Aspbr1_135712
Anigcbs51388_145246678
Akaw_358369912
Aspfo1_164757
Anig_fge1_pg_C_9000331.1
Pench1_32369
Afla_03239
Aory_AO090012000330
Aspac1_62723
Ater_07453
Aspve1_180655
Aspsy1_51627
Anid_AN9369
Aory_AO090120000007
Afla_08051
Acar_17097
Aspfo1_135356
Akaw_358373913
Anigcbs51388_145250645
Anig_fge1_pm_C_6000159.1
Aspbr1_131585
Ater_06316
Anid_AN6882
Aspsy1_170071
Aspve1_58021
Afum_Afu2g00120
Phieu1_HMPREF154106177T0
Claps1_A1O508631T0
Claca1_G64706586T0
Claye1_A1O705956T0
Exoaq1_A1O905366T0
0.5

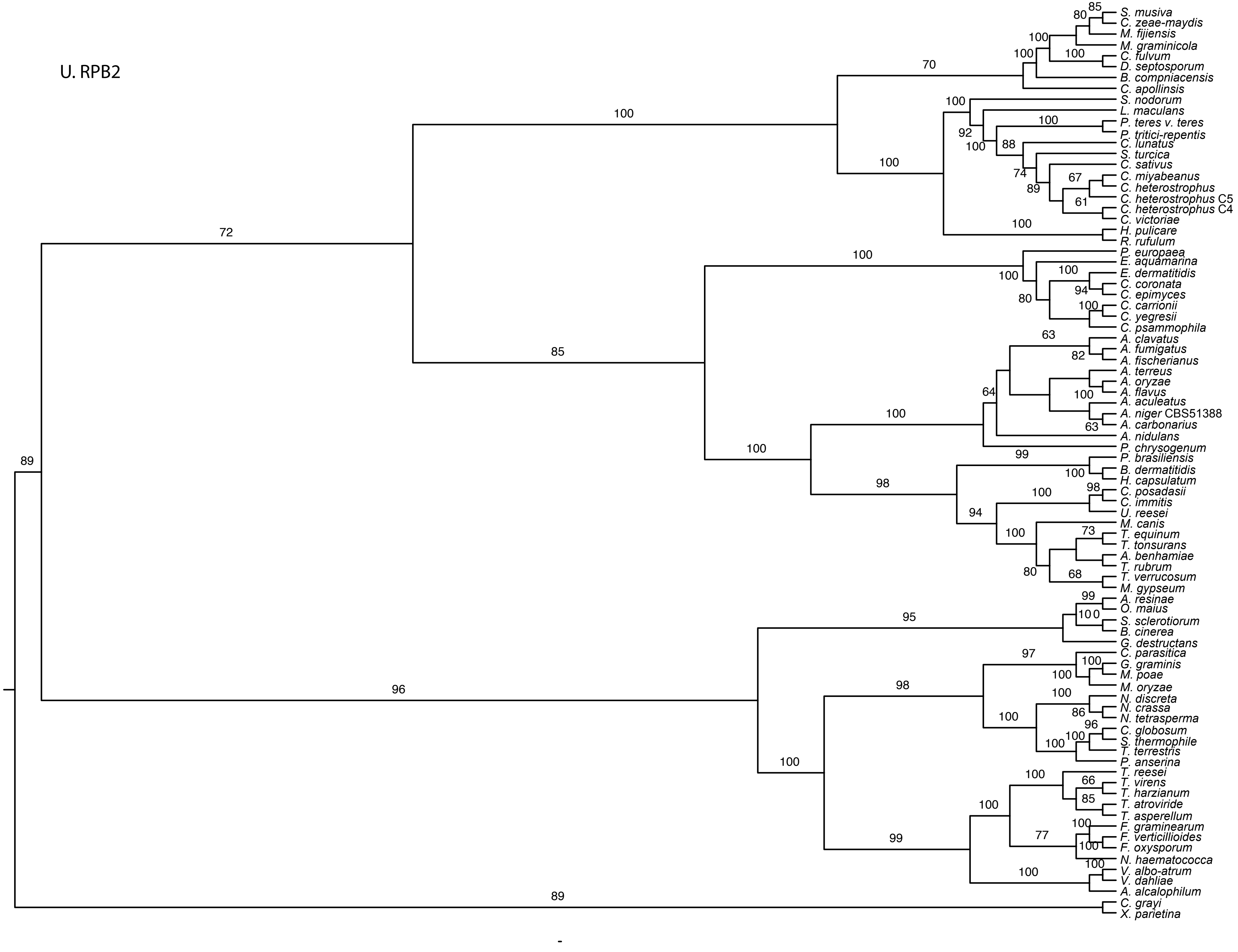

U. RPB2
S. musiva
C. zeae-maydis
M. fijiensis
M. graminicola
C. fulvum
D. septosporum
B. compniacensis
C. apollinsis
S. nodorum
L. maculans
P. teres v. teres
P. tritici-repentis
C. lunatus
S. turcica
C. sativus
C. miyabeanus
C. heterostrophus
C. heterostrophus C5
C. heterostrophus C4
C. victoriae
H. pulicare
R. rufulum
P. europaea
E. aquamarina
E. dermatitidis
C. coronata
C. epimyces
C. carrionii
C. yegresii
C. psammophila
A. clavatus
A. fumigatus
A. fischerianus
A. terreus
A. oryzae
A. flavus
A. aculeatus
A. niger CBS51388
A. carbonarius
A. nidulans
P. chrysogenum
P. brasiliensis
B. dermatitidis
H. capsulatum
C. posadasii
C. immitis
U. reesei
M. canis
T. equinum
T. tonsurans
A. benhamiae
T. rubrum
T. verrucosum
M. gypseum
A. resinae
O. maius
S. sclerotiorum
B. cinerea
G. destructans
C. parasitica
G. graminis
M. poae
M. oryzae
N. discreta
N. crassa
N. tetrasperma
C. globosum
S. thermophile
T. terrestris
P. anserina
T. reesei
T. virens
T. harzianum
T. atroviride
T. asperellum
F. graminearum
F. verticillioides
F. oxysporum
N. haematococca
V. albo-atrum
V. dahliae
A. alcalophilum
C. grayi
X. parietina

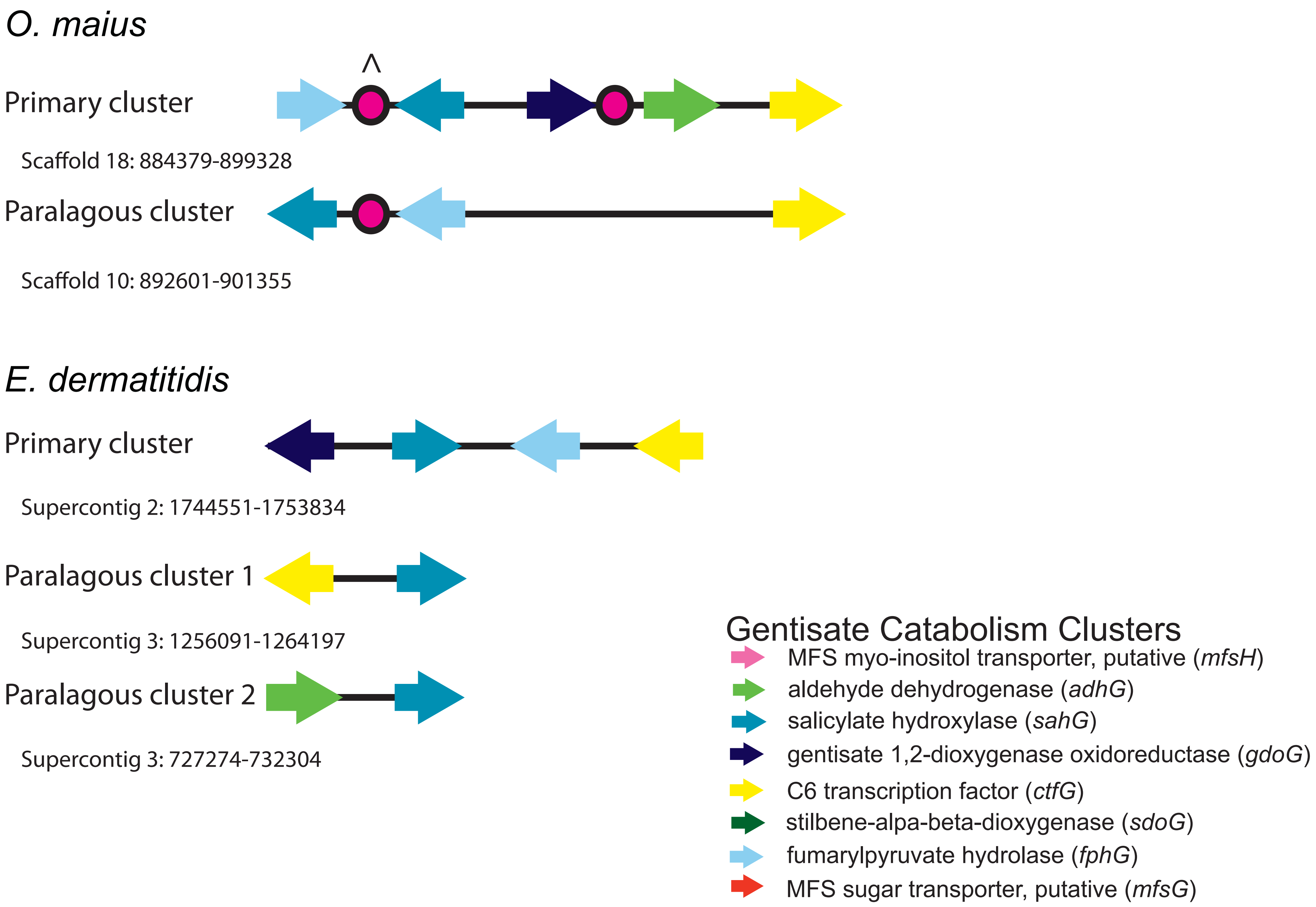
O. maius
Primary cluster
^
Scaffold 18: 884379-899328
Paralagous cluster
Scaffold 10: 892601-901355
E. dermatitidis
Primary cluster
Supercontig 2: 1744551-1753834
Paralagous cluster 1
Supercontig 3: 1256091-1264197
Paralagous cluster 2
Supercontig 3: 727274-732304
Gentisate Catabolism Clusters
MFS myo-inositol transporter, putative (mfsH)
aldehyde dehydrogenase (adhG)
salicylate hydroxylase (sahG)
gentisate 1,2-dioxygenase oxidoreductase (gdoG)
C6 transcription factor (ctfG)
stilbene-alpa-beta-dioxygenase (sdoG)
fumarylpyruvate hydrolase (fphG)
MFS sugar transporter, putative (mfsG)

A. Bacterial gentisate 1,2-dioxygenase alignment Logo

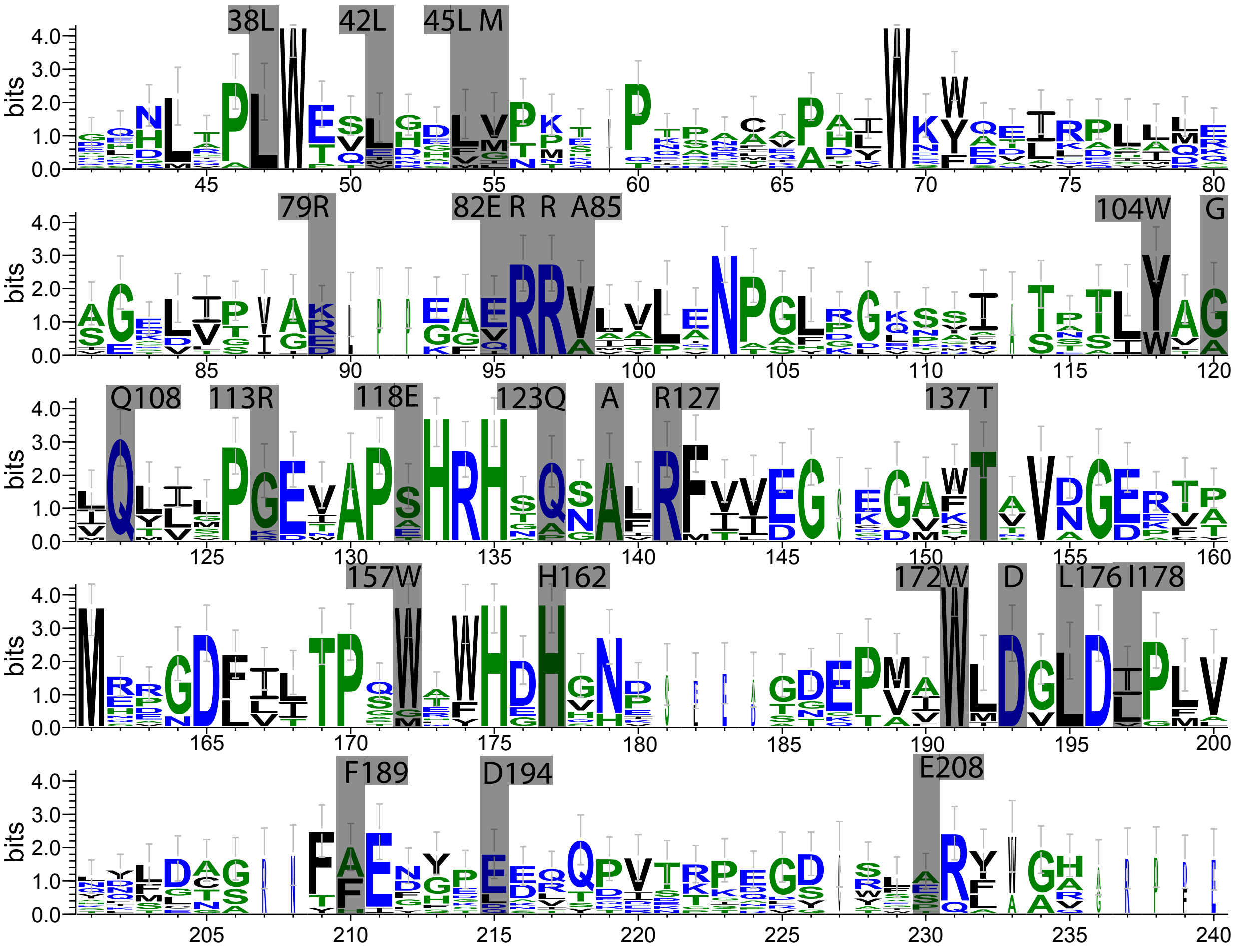

B. Fungal gentisate 1,2-dioxygenase alignment Logo

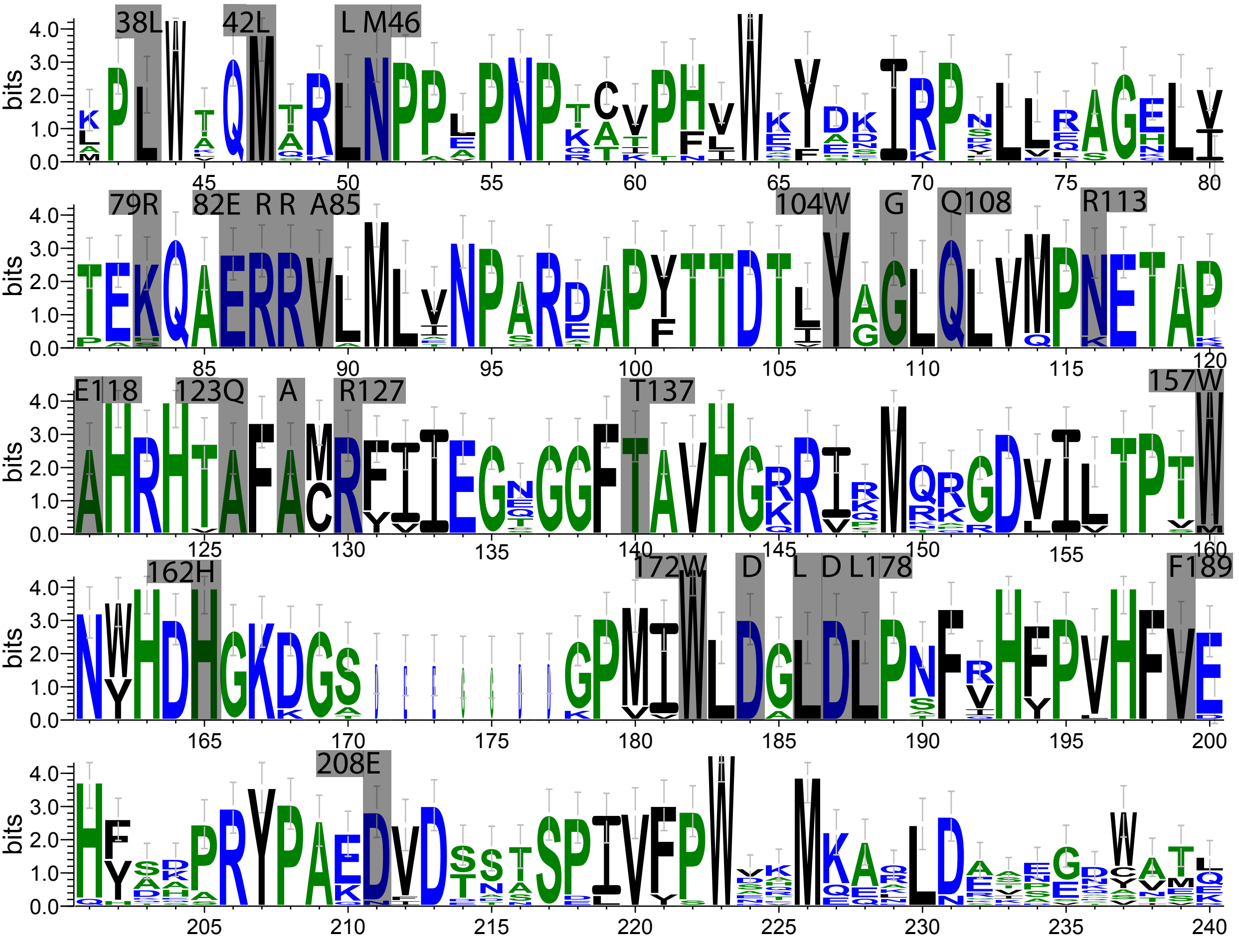

A

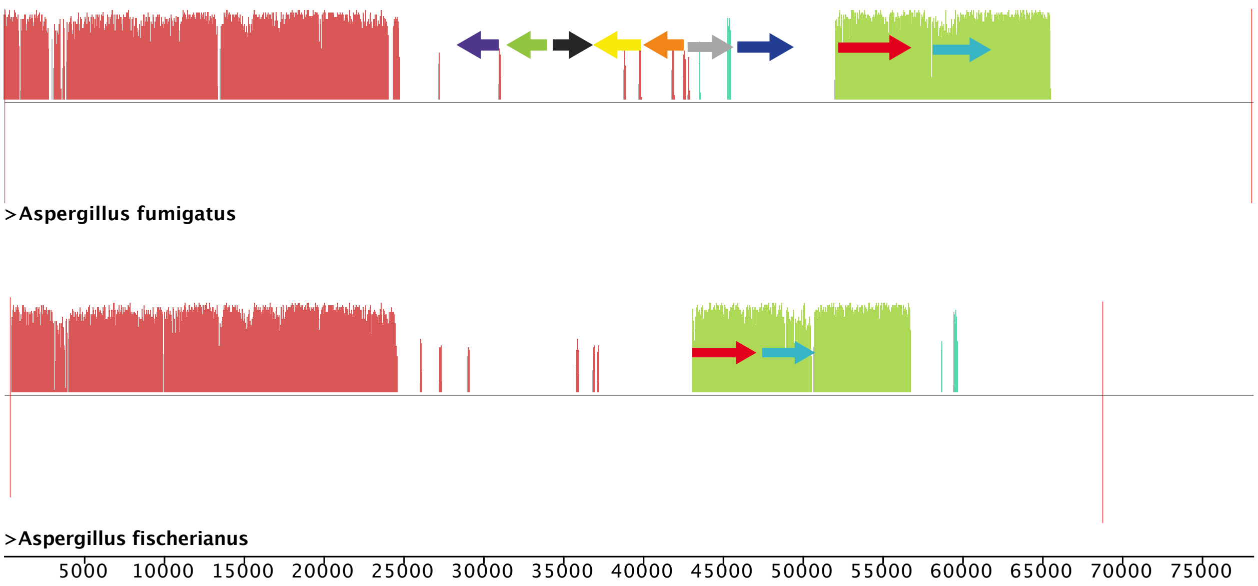
>Aspergillus fumigatus
>Aspergillus fischerianus
5000
10000
15000
20000
25000
30000
35000
40000
45000
50000
55000
60000
65000
70000
75000

B

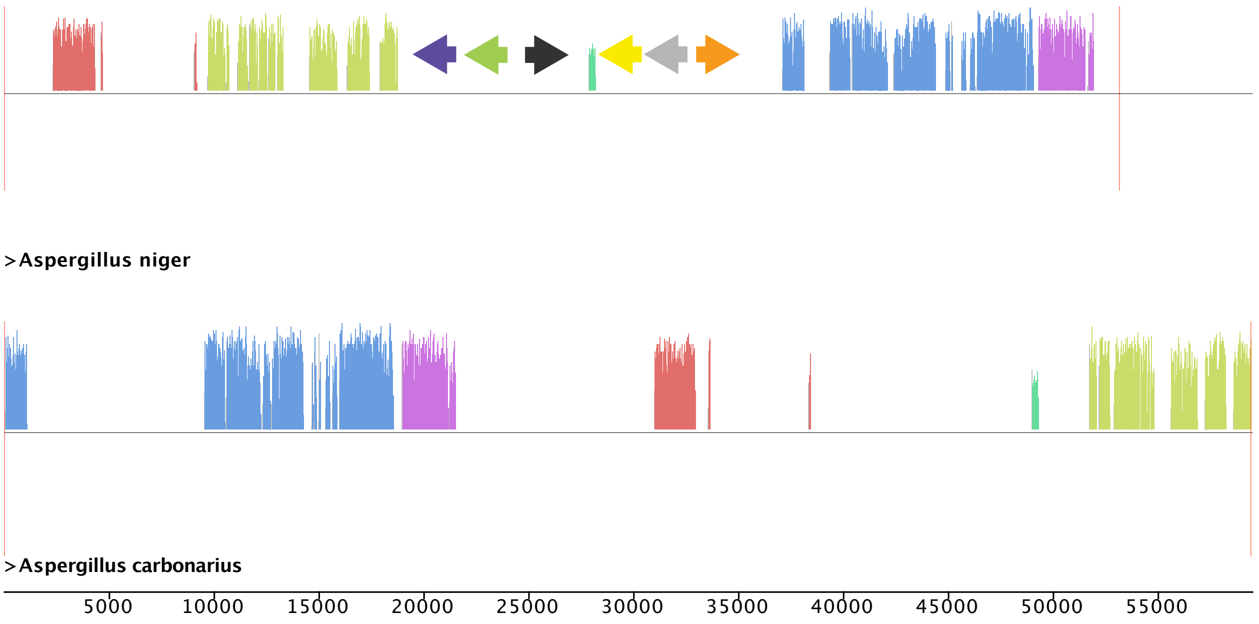
>Aspergillus niger
>Aspergillus carbonarius
5000
10000
15000
20000
25000
30000
35000
40000
45000
50000
55000

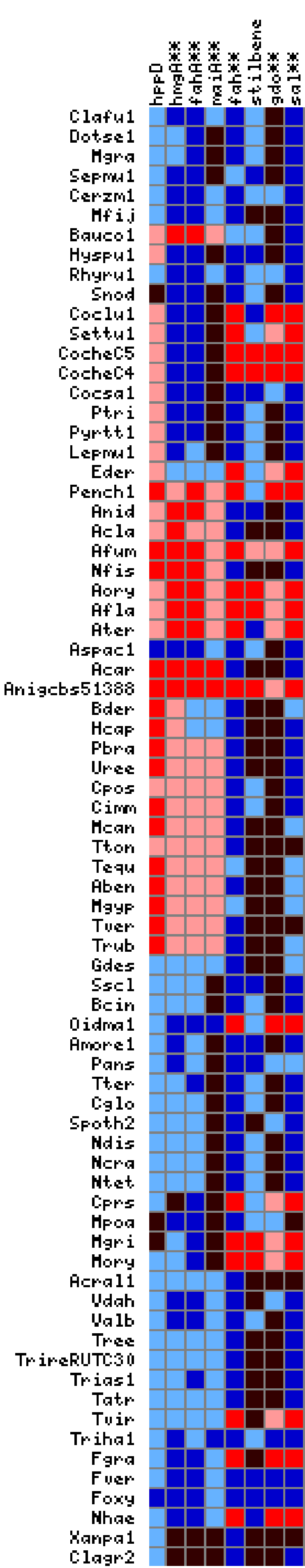
hpfD
hmgA**
fahA**
maiA**
fah**
stilbene
gdo**
sal**
Clafu1
Dotse1
Mgra
Sepmu1
Cerzm1
Mfij
Bauco1
Hyspu1
Rhyru1
Snod
Coclu1
Settu1
CocheC5
CocheC4
Cocsa1
Ptri
Pyrtt1
Lepmu1
Eder
Pench1
Anid
Acla
Afum
Nfis
Aory
Afla
Ater
Aspac1
Acar
Anigcbs51388
Bder
Hcap
Pbra
Uree
Cpos
Cimm
Mcan
Tton
Tequ
Aben
Mgyp
Tver
Trub
Gdes
Sscl
Bcin
Oidma1
Amore1
Pans
Tter
Cglo
Spoth2
Ndis
Ncra
Ntet
Cpre
Mpoa
Mgri
Mory
Acral1
Vdah
Valb
Tree
TrireRUTC30
Trias1
Tatr
Tvir
Triha1
Fgra
Fver
Foxy
Nhae
Xanpa1
Clagr2

Table S1. Genomes analyzed in this study.

Genome	Genome Code	Ecology	TD	GC +*sdoG*	GC -sdoG
**Genomes in Figure 2**					
Acremonium alcalophilum	Acral1	sludge of pig manure compost	-	-	-
Amorphotheca resinae ATCC 22711	Amore1	jet fuel	-	-	-
Arthroderma benhamiae CBS 112371	Aben	human dermatophyte	+	-	-
Aspergillus aculeatus	Aspac1	soil, tomato, grape	+	-	-
Aspergillus carbonarius ITEM 5010	Acar	Grape berry, Brindis, Apulia, Italy.	+	-	-
Aspergillus clavatus NRRL 1	Acla	soil, manure, wheat, barley, maize	+	-	-
Aspergillus flavus NRRL 3357	Afla	corn, peanuts, cottonseed	+	+	-
Aspergillus fumigatus Af293	Afum	corn silage, lung tissue	+	+	-
Aspergillus nidulans FGSC A4	Anid	laboratory strain	+	-	-
Aspergillus niger	Anig	fruit, nuts, peanuts, cereal, meat, cheese	+	+	-
Aspergillus oryzae RIB40/ATCC 42149	Aory	cereal	+	+	-
Aspergillus terreus	Ater	soil, stored cereal	+	+	-
Baudonia compniacensis UAMH 10762	Bauco1	Exterior surfaces around distilleries	+	-	-
Blastomyces dermatitidis	Bder	soil, pathogen of mammals	+	-	-

Botrytis cinerea	Bcin	pathogen of platns: vegetables, fruits, shrubs, trees, flowers, weeds	-	-	-
Capronia coronata cbs 617.96	Capco1	waterborne fungus associated with plants	-	-	-
Capronia epimyces cbs 606.96	Capep1	hyperparasite associated with plants	+	-	-
Cercospora zeae-maydis SCOH1-5	Cerzm1	pathogen of maize	-	-	-
Chaetomium globosum CBS 148.51	Cglo	decaying plant material	-	-	-
Cladonia grayi	Cgra	lichen	-	-	-
Cladophialophora psammophila cbs 110553	Claps1	hydrocarbon degrader	+	-	+
Cladophialophora yegresii cbs 114405	Claye1	epiphyte of cactus spines	+	-	+
Cladosporium fulvum	Clafu1	pathogen of tomato	-	-	-
Coccidioides immitis	Cimm	pathogen of humans	+	-	-
Coccidioides posadasii	Cpos	pathogen of humans	+	-	-
Cochliobolus heterostrophus C4	CocheC4	pathogen of maize	+	+	-
Cochliobolus heterostrophus C5	CocheC5	pathogen of maize	+	+	-
Cochliobolus lunatus m118	Coclu2	pathogen of sorghum; opportunistic pathogen of humans	+	+	-
Cochliobolus sativus ND90Pr	Cocsa1	Tester for mating	+	-	-

Coniosporium apollinis cbs 100218	Conap1	type A rock-inhabiting fungus	-	-	-
Cryphonectria parasitica EP 155	Cprs	pathogen of chestnut trees	-	-	+
Dothistroma septosporum NZE10	Dotse1	pathogen of pine	-	-	-
Exophiala aquamarina cbs 119918	Exoaq1	marine animal pathogen	+	-	-
Exophiala dermatitidis UT8656	Eder	pathogen of humans	+	-	+
Fusarium graminearum	Fgra	pathogen of wheat and barley; endophyte	-	-	+
Fusarium oxysporum	Foxy	pathogen of fruit; endophyte	-	-	-
Fusarium verticillioides 7600	Fver	pathogen of maize; endophyte	-	-	-
Gaeumannomyces graminis var. tritici r3-111a-1	Gaegr1	pathogen of wheat and barley	-	-	+
Geomyces destructans 20631-21	Gdes	brown bat	-	-	-
Histoplasma capsulatum	Hcap	bird/bat feces; pathogen of mammals	+	-	-
Hysterium pulicare	Hyspu1	Ascospore from Betula nigra	+	-	-
Leptosphaeria maculans	Lepmu1	pathogen of oilseed rape	+	-	-
Magnaporthe oryzae 70-	Mory	pathogen of rice	-	+	-

15(MG6)					
Magnaporthe poae ATCC 64411	Mpoa	pathogen of rice	-	-	-
Microsporum canis CBS 113480	Mcan	Head of 3-year-old patient, Wurzburg, Germany.	+	-	-
Microsporum gypseum 118893	Mgyp	Skin of patient, Uganda.	+	-	-
Mycospharella fijiensis 10313	Mfij	pathogen of banana	-	-	-
Mycospharella graminicola 11395	Mgra	pathogen of wheat	-	-	-
Nectria haematococca MPV1	Nhae	plant pathogen, wide host range	-	-	+
Neosartorya fischeri NRRL 181	Nfis	soil, canned food	+	-	-
Neurospora crassa OR74A	Ncra	decaying plant material	-	-	-
Neurospora discreta FHSC 8579 matA	Ndis	forest fire remains (trees)	-	-	-
Neurospora tetrasperma FGSC 2508 matA	Ntet	decaying plant material	-	-	-
Oidiodendron maius Zn	Oidma1	endophyte of ericaceous plants	-	-	+
Paracoccidioides brasiliensis Pb01	Pbra	human cervical lymph node, Belo Horizonte, Minas Gerais, Brazil	+	-	-
Penicillium chrysogenum Wis. 54-1255	Pench1	industrial strain	+	-	+
Podospora anserina	Pans	herbivore dung	-	-	-
Pyrenophora teres f. teres	Pyrtt1	pathogen of barley	+	-	-

Pyrenophora tritici-repentis	Ptri	Pathogen of grass	+	-	-
Rhytidhysteron rufulum	Rhyru1	saprobic with diverse habitats; wood of dicotyledonous plants	+	-	-
Sclerotinia sclerotiorum ATCC 18683	Sscl	moist soils, pathogen of wide variety of plants	-	-	-
Septoria musiva S02202	Sepmu1	pathogen of poplars	-	-	-
Setosphaeria turcica Et28A	Settu1	pathogen of maize	+	+	-
Sporotrichum thermophile	Spoth2	decaying plant material	-	-	-
Stagnospora nodorum SN15	Snod	pathogen of wheat	+	-	-
Thielavia terrestris NRRL 8126	Tter	thermophile	-	-	-
Trichoderma asperellum CBS 433.97	Trias1	soil, endophyte, mycoparasite	-	-	-
Trichoderma atroviride IMI 206040	Tatr	soil, endophyte, mycoparasite	-	-	-
Trichoderma harzianum	Triha1	soil, endophyte, mycoparasite, wood decay	-	-	-
Trichoderma reesei	Tree	soil, wood decay	-	-	-
Trichoderma reesei RUT C-30	TrireRUTC30	soil, wood decay	-	-	-
Trichoderma virens Gv29-8	Tvir	soil, endophyte, mycoparasite	-	-	+
Trichophyton equinum CBS 127.97	Tequ	horse	+	-	-
Trichophyton rubrum CBS	Trub	Patient with	+	-	-

118892		onychomycosis, Germany.			
Trichophyton tonsurans CBS 112818	Tton	cheek of man, Montreal, Quebec, Canada.	+	-	-
Trichophyton verrucosum HKI0517	Tver	bovine	+	-	-
Uncinocarpus reesii 1704	Uree	soil	+	-	-
Verticillium albo-atrum	Valb	soil, pathogen of alfalfa, cotton, hops	-	-	-
Verticillium dahliae	Vdah	soil, pathogen of wide variety of plants	-	-	-
Xanthoria parietina 46-1	Xanpa1	lichen	-	-	-
**Other genomes investigated**					
Agaricus bisporus var. bisporus	Abibi				
Agaricus bisporus var. burnettii	Abibu				
Acidomyces richmondensis	Aciri1iso				
Allomyces macrogynus	Amac				
Aspergillus niger	Anigcbs51388				
Ascoidea rubescens NRRL Y17699	Ascru1				
Aspergillus brasiliensis	Aspbr1				
Aspergillus acidus	Aspfo1				
Aspergillus sydowii	Aspsy1				
Aspergillus versicolor	Aspve1				
Auricularia delicata SS-5	Aurde1				
Aureobasidium pullulans var. subglaciale EXF-2481	Aurpuvarsub1				

Babjeviella inositovora NRRL Y-12698	Babin1
Batrachochytrium dendrobatidis	Bden
Bjerkandera adusta	Bjead1
Botryobasidium botryosum	Botbo1
Candida albicans	Calb
Candida arabinofermentans NRRL YB-2248	Canar1
Candida caseinolytica Y-17796	Canca1
Candida tanzawaensis NRRL Y-17324	Canta1
Candida tenuis NRRL Y-1498	Cante1
Catenaria anguillulae PL171	Catan1
Coprinopsis cinerea	Ccin
Candida glabrata	Cgla138
Candida guilliermondii	Cgui
Cochliobolus heterostrophus	Chet
Cladophialophora carrionii cbs 160.54	Claca1
Cladonia grayi(newer annotation)	Clagr2
Candida lusitaniae	Clus
Cryptococcus neoformans	Cneb
Cryptococcus gattii	Cneg
Cryptococcus neoformans	Cnej
Cryptococcus neoformans var. grubii	Cneo
Cryptococcus gattii	Cnew

Cochliobolus carbonum 26-R-13	Cocca1
Cochliobolus miyabeanus ATCC 44560	Cocmi1
Cochliobolus victoriae FI3	Cocvi1
Coemansia reversa NRRL 1564	Coere1
Conidiobolus coronatus NRRL28638	Conco1
Coniophora puteana	Conpu1
Candida parapsilosis	Cpar
Cronartium quercuum f. sp. fusiforme G11	Croqu1
Ceriporiopsis subvermispora	Csub
Candida tropicalis	Ctro
Dacryopinax sp. DJM 731 SSP1	Dacsp1
Dekkera bruxellensis CBS 2499	Dekbr2
Debaryomyces hansenii	Dhan
Dichomitus squalens	Dicsq1
Encephalitozoon cuniculi	Ecun
Eremothecium cymbalariae	Ecym
Eremothecium gossypii	Egos
Encephalitozoon intestinalis ATCC 50506	Eint
Fomitiporia mediterranea	Fomme1
Fomitopsis pinicola SS1	Fompi1
Galerina marginata	Galma1
Gonapodya prolifera	Ganpr1
Gloeophyllum trabeum	Glotr1
Gymnopus luxurians	Gymlu1

Heterobasidion annosum	Hann
Hanseniaspora valbyensis NRRL Y-1626	Hanva1
Hebeloma cylindrosporum h7	Hebcy1
Hansenula polymorpha	Hpol
Hydnomerulius pinastri	Hydpi1
Hydnomerulius pinastri	Hydpi2
Hyphopichia burtonii NRRL Y-1933	Hypbu1
Hypholoma sublateritium	Hypsu1
Jaapia argillacea	Jaaar1
Kluyveromyces lactis	Klac
Kluyveromyces thermotolerans	Kthe
Kluyveromyces waltii	Kwal
Laccaria bicolor	Lbic
Lodderomyces elongisporus	Lelo
Lipomyces starkeyi NRRL Y-11557	Lipst1
Mucor circinelloides	Mcir
Metschnikowia bicuspidata NRRL YB-4993	Metbi1
Malassezia globosa	Mglo
Mixia osmundae IAM 14324	Mixos1
Melampsora laricis-populina	Mlar
Nadsonia fulvescens var. elongata DSM 6958	Nadfu1
Paxillus involutus ATCC 200175	Paxin1
Phycomyces blakesleeanus	Pbla
Phanerochaete chrysosporium	Pchr

Puccinia graminis f. sp. Tritici	Pgra
Phanerochaete carnosa HHB-10118-Sp	Phaca1
Phialophora europaea cbs 101466	Phieu1
Phlebiopsis gigantea	Phlgi1
Pichia membranifaciens	Picme2
Piloderma croceum F 1598	Pilcr1
Piromyces sp. E2	PirE2
Pisolithus microcarpus 441	Pismi1
Pisolithus tinctorius Marx 270	Pisti1
Plicaturopsis crispa	Plicr1
Postia placenta MAD-698-R-SB12	PosplRSB12
Pleurotus ostreatus	Post
Pichia pastoris GS115	Ppas
Postia placenta	Ppla
Pichia stipitis	Psti
Punctularia strigosozonata	Punst1
Puccinia triticina 1-1BBBD Race 1	Putr
Rhodotorula graminis strain WP1	Rhoba1
Rhizopus oryzae	Rory
Saitoella complicata NRRL Y-17804	Saico1
Saccharomyces bayanus	Sbay
Saccharomyces castellii	Scas
Saccharomyces cerevisiae	Scer

Scleroderma citrinum Foug A	Sclci1
Schizophyllum commune	Scom
Sebacina vermifera MAFF 305830	Sebve1
Serpula lacrymans S7	SerlaS7
Serpula lacrymans var shastensis SHA21-2	Serlavarsha1
Schizosaccharomyces japonicus	Sjap
Saccharomyces kluyveri	Sklu
Saccharomyces kudriavzevii	Skud
Serpula lacrymans	Slac
Saccharomyces mikatae	Smik
Schizosaccharomyces octosporus	Soct
Schizosaccharomyces cryophilus	Soyt
Spathaspora passalidarum NRRL Y-27907	Spapa3
Saccharomyces paradoxus	Spar
Sphaerobolus stellatus	Sphst1
Schizosaccharomyces pombe	Spom
Spizellomyces punctatus	Spun
Sporobolomyces roseus	Sros
Stereum hirsutum FP-91666 SS1	Stehi1
Tremella mesenterica Fries	Tmes
Tetrapisispora phaffii CBS 4417	Tpha
Trametes versicolor	Trave1
Tulasnella calospora AL13/4D	Tulca1
Ustilago maydis	Umay

Vanderwaltozyma_polyspora	Vpol
Wickerhamomyces anomalus	Wican1
Wolfiporia cocos MD-104 SS10	Wolco1
Yarrowia lipolytica	Ylip
Zygosaccharomyces rouxii	Zrou

Table S2. Comparison of function-related residues among gentisate 1,2 dioxygenase homologs in fungi and bacteria.

function	*P. salicylatoxidans*	Bacteria	*A. fumigatus*
Active site, conserved; stabilizes 4°	Leu(38)	Leu	Leu(31)
Closure of active site	Leu(42)	L/E/I/M/V	Met
Active site	Leu(45)	L/F/V/M	Leu(38)
Active site	Met(46)	V/M/G/I/L	Asn(39)
Closure of active site	Arg(79)	K/R/E/D	Lys
Active site	Glu(82)	E/V/Q/I	Glu(74)
Active site; closure of AS; stabilizes 4°	Arg(83)	Arg	Arg(75)
	Arg(84)	Arg	Arg(76)
Active site; stabilizes 4°	Ala(85)	Val>Ala	Val(77)
Active site	Trp(104)	Tyr>Trp	Tyr(95)
Substrate selection/binding	Gly(106)	Gly>>Ala	Gly(97)
Active site, conserved	Gln(108)	Gln	Gln(99)
	Arg(113)	Gly>K/R	Asn(104)
Active site closure	Glu(118)	S/A/E/T	Ala
Active site	Gln(123)	Q>A/P	Ala(114)
Active site; Coordination of metal ion/substrate	Ala(125)	Ala	Ala(116)
Active site, conserved	Arg(127)	Arg	Arg(118)
Active site	Thr(137)	Thr	Thr(128)
Hydrophobic shielding of active site	Trp(157)	Trp>G/M	Trp(148)
Active site, conserved	His(162)	His	His(153)
Active site, conserved	Trp(172)	Trp	Trp(163)
Active site, conserved	Asp(174)	Asp	Asp(165)
Active site, conserved	Leu(176)	Leu	Leu(167)
Active site	Ile(178)	I/L/V	Ile(169)
Stabilizes 4°	Phe*(189)	Y/G/H/F/S	Val(180)
Closure of active site	Asp(194)	E/L/D/Q	-
	Gln(208)	A/E/S/P/T	
